# Neurite density imaging in amygdala nuclei reveals interindividual differences in neuroticism

**DOI:** 10.1002/hbm.25775

**Published:** 2022-01-20

**Authors:** Caroline Schlüter, Christoph Fraenz, Patrick Friedrich, Onur Güntürkün, Erhan Genç

**Affiliations:** ^1^ Department of Biopsychology, Institute of Cognitive Neuroscience, Faculty of Psychology Ruhr University Bochum Bochum Germany; ^2^ Department of Psychology and Neurosciences Leibniz Research Centre for Working Environment and Human Factors (IfADo) Dortmund Germany; ^3^ Institute of Neuroscience and Medicine, Brain & Behaviour (INM‐7), Research Centre Jülich Jülich Germany

**Keywords:** amygdala nuclei, depression, neurite density, neuroticism, NODDI

## Abstract

Neuroticism is known to have significant health implications. While previous research revealed that interindividual differences in the amygdala function are associated with interindividual differences in neuroticism, the impact of the amygdala’s structure and especially microstructure on variations in neuroticism remains unclear. Here, we present the first study using NODDI to examine the association between the in vivo microstructural architecture of the amygdala and neuroticism at the level of neurites. We, therefore, acquired brain images from 221 healthy participants using advanced multi‐shell diffusion‐weighted imaging. Because the amygdala comprises several nuclei, we, moreover, used a high‐resolution T1 image to automatically segment the amygdala into eight different nuclei. Neuroticism and its facets have been assessed using the NEO‐PI‐R. Finally, we associated neuroticism and its facets with the volume and microstructure of the amygdala nuclei. Statistical analysis revealed that lower neurite density in the lateral amygdala nucleus (La) was significantly associated with higher scores in depression, one of the six neuroticism facets. The La is the sensory relay of the amygdala, filtering incoming information based on previous experiences. Reduced neurite density and related changes in the dendritic structure of the La could impair its filtering function. This again might cause harmless sensory information to be misevaluated as threatening and lead to the altered amygdala responsivity as reported in previous studies investigating the functional correlates of neuroticism and neuroticism‐related disorders like depression.

## Graphical abstract

We present the first study using NODDI to examine the association between the in vivo microstructural architecture of the amygdala and neuroticism at the level of neurites. We, therefore, acquired brain images from 221 healthy participants using advanced multi‐shell diffusion‐weighted imaging. Because the amygdala comprises several nuclei, we, moreover, used a high‐resolution T1 image to automatically segment the amygdala into eight different nuclei. Neuroticism and its facets have been assessed using the NEO‐PI‐R. Finally, we associated neuroticism and its facets with the volume and microstructure of the amygdala nuclei. Statistical analyses revealed that lower neurite density in the lateral amygdala nucleus (La) was significantly associated with higher scores in depression, one of the six neuroticism facets.

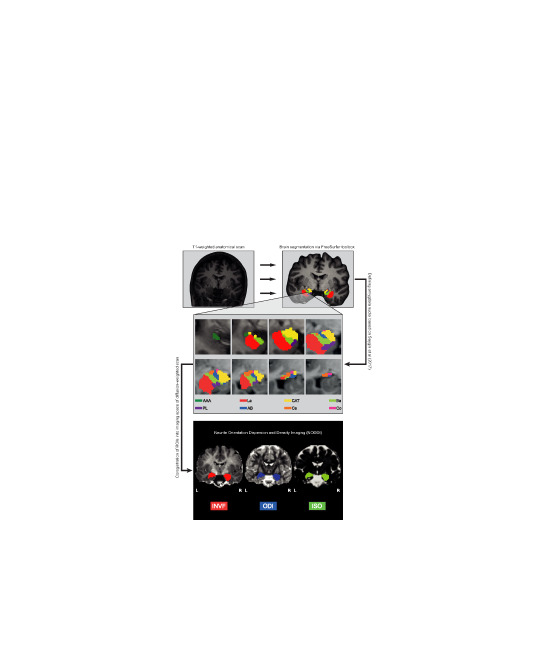

## 1 INTRODUCTION

Due to its well‐documented importance to our physical and especially mental health, neuroticism is among the best‐studied personality traits. Comprising the overall tendency to experience negative emotions, as well as an attentional bias toward aversive stimuli (Schweckendiek, Stark, & Klucken, 2016; Shackman et al., 2016) neuroticism is considered to be a general risk factor for all kinds of mental health issues, such as sleep problems, major depressive disorder, generalized anxiety disorder, or even schizophrenia (Hintsanen et al., 2014; Lahey, 2009; Ormel, Jeronimus, et al., 2013). For instance, longitudinal studies on the relationship between personality and mental health could show that neuroticism not only significantly influences the propensity for a mental disorder, like major depression (Kendler, Gatz, Gardner, & Pedersen, 2006), but is also associated with increased comorbidity (Hengartner, Kawohl, Haker, Rössler, & Ajdacic‐Gross, 2016). Apart from the fact that neuroticism favors the onset of mental health issues, a longitudinal study by Cuijpers et al. (2010) revealed that the economic costs of neuroticism itself exceed those of common mental disorders by far.

Due to the adverse influence on our well‐being (Lahey, 2009) and our national economy (Cuijpers et al., 2010), neuroscientists have become increasingly involved in exploring the mechanisms that underlie neuroticism. One of the first scientists working on the biological mechanisms of neuroticism was Hans Eysenck. Eysenck related individual differences in neuroticism to lower activation thresholds in brain areas that process and regulate emotions (Eysenck, 1967; Eysenck & Eysenck, 1985; Servaas et al., 2013). Thus, it was hypothesized that individuals who score high on neuroticism are more likely to become emotionally aroused when facing an unpleasant stimulus than individuals with low neuroticism scores (Eysenck, 1967; Eysenck & Eysenck, 1985). Plenty of the previous studies on the biological basis of neuroticism were conducted in the spirit of Eysenck, focusing on the functional activity of areas that are related to emotional processing and regulation (Servaas et al., 2013). A large number of these studies targeted the amygdala as their region of interest (Brück, Kreifelts, Kaza, Lotze, & Wildgruber, 2011; Canli, 2004; Canli et al., 2001; Everaerd, Klumpers, van Wingen, Tendolkar, & Fernández, 2015; Haas, Omura, Constable, & Canli, 2007; Schweckendiek et al., 2016; Servaas et al., 2013). Here, researchers examined the functional amygdala activity in response to stimuli of different valence (Servaas et al., 2013). For instance, Brück et al. (2011) found that higher neuroticism scores lead to higher amygdala activity while listening to emotional prosody compared to vowels or emotional semantics. Further, Haas et al. (2007) found that neuroticism correlated positively with functional amygdala activity when the participant was confronted with emotional conflict. A study by Schweckendiek et al. (2016) showed that individual differences in neuroticism are related to differences in amygdala activity during appetitive conditioning. Thus, not only how we process emotional stimuli but also the way we learn through reward or punishment is related to our personality. This often‐reported relationship between enhanced amygdala reactivity to an emotional stimulus and neuroticism seems to be particularly pronounced under stressful conditions (Everaerd et al., 2015; Shackman et al., 2016). However, the results on the functional reactivity of the amygdala have been partly inconsistent and could not be confirmed in all studies (Everaerd et al., 2015; Servaas et al., 2013). Regarding the structural correlates of neuroticism, a similar picture emerges (Allen & DeYoung, 2017). While a promising study by Holmes et al. (2012), with more than 1,000 subjects, shows a significant, albeit weak, correlation between amygdala volume and neuroticism, several other studies have not (Avinun, Israel, Knodt, & Hariri, 2019; DeYoung, 2010; Fuentes et al., 2012; Liu et al., 2013; Owens et al., 2019; Wright et al., 2006). The causes for the inconsistency of findings appear to be manifold. They might be due to the rather small sample sizes usually found in personality neuroscience (Allen & DeYoung, 2017; Mar, Spreng, & DeYoung, 2013), as well as to the assessment of neuroticism used in the respective studies (Servaas et al., 2013). In this context, Ormel and colleagues argued that neuroticism is a heterogeneous construct measured with questionnaires including different, partly opposing, facets (Ormel, Bastiaansen, et al., 2013; Ormel, Jeronimus, et al., 2013). Thus, the authors propose that future research investigating the biological mechanisms behind neuroticism would benefit from deconstructing neuroticism into lower‐order facets (Ormel, Jeronimus, et al., 2013).

Moreover, the amygdala is also not a unified structure, but a cluster of nuclei that differ significantly in both structure and function (Duvarci & Pare, 2014; Yilmazer‐Hanke, 2012). From this point of view, it seems reasonable not to examine the amygdala as a whole, but instead investigate the role of the amygdala’s nuclei. This assumption is confirmed by a variety of animal studies showing that the individual amygdala nuclei take a different part in the processing of emotional stimuli (Büchel & Dolan, 2000; LeDoux, Cicchetti, Xagoraris, & Romanski, 1990; Martin‐Soelch, Linthicum, & Ernst, 2007; Yilmazer‐Hanke, 2012). For example, it has been shown that the basolateral amygdala nucleus is associated with the evaluation of the unconditioned stimulus in classical conditioning. Lesion of the basolateral nucleus impairs the evaluation of a possible threat or reward, which in turn hinders the conditioning process (Hatfield, Han, Conley, Gallagher, & Holland, 1996; Setlow, Gallagher, & Holland, 2002). Beyond that, the lateral nucleus (La) of the amygdala is considered to be the sensory input station of the amygdala complex (Yilmazer‐Hanke, 2012). It receives information from sensory and associative brain areas, evaluates them, and forwards them to other amygdala nuclei (Yilmazer‐Hanke, 2012). Thus, regarding emotional processing the La is considered to combine sensory stimuli with emotive value ‐ a function that is especially critical in classical conditioning (Duvarci & Pare, 2014; LeDoux et al., 1990; Sigurdsson, Doyère, Cain, & LeDoux, 2007). These studies strongly suggest investigating the amygdala at nuclei level when examining the neural correlates of neuroticism.

To study the structural and functional nature of the amygdala at nuclei level, previous studies have typically conducted manual parcellations (A. Aghamohammadi‐Sereshki, Huang, Olsen, & Malykhin, 2018; Entis, Doerga, Barrett, & Dickerson, 2012). However, there are some downsides to this methodological approach. First, standard and even ultra‐high‐resolution scans do not allow for the human eye to distinguish all amygdala nuclei. Thus, manual techniques commonly yield only a small number of amygdala segments (A. Aghamohammadi‐Sereshki et al., 2018; Entis et al., 2012). Second, manual segmentation is rater‐dependent and time consuming (A. Aghamohammadi‐Sereshki et al., 2018). Although previous studies report high inter‐rater reliabilities, they are usually based on few raters and small‐sized samples (A. Aghamohammadi‐Sereshki et al., 2018; Entis et al., 2012). For large samples, however, manual segmentation appears to be inappropriate. A recent FreeSurfer atlas, developed by Saygin et al. (2017), offers a solution to these issues. The atlas allows for a rater‐independent automatic segmentation of nine amygdala nuclei from structural magnetic resonance images. The automatic parcellation makes visual nuclei identification superfluous and enables amygdala segmentation even at lower resolutions (Saygin et al., 2017). This atlas‐based automated parcellation does justice to the different structures and functions of the amygdala, increases comparability between studies, and enables more researchers to investigate the amygdala on the level of nuclei. Despite this new method, there have been no studies considering the role of different amygdala nuclei regarding interindividual differences in neuroticism until now.

Beyond the functional and macrostructural brain correlates of neuroticism, postmortem examinations of patients with neuroticism‐related disorders revealed, that changes in the microstructure of the amygdala might found the neural basis of neuroticism‐related behaviors (Altshuler et al., 2010; Berretta, Pantazopoulos, & Lange, 2007; Bowley, Drevets, Öngür, & Price, 2002; Dossi, Vasile, & Rouach, 2018). Here, again, it is crucial to examine the microstructural architecture of the different amygdala nuclei distinctly. For example, an interesting postmortem study of Schumann and Amaral (2006) analyzed the neuron number in the amygdala of patients with autism finding that, even though the amygdala volume, in general, does not vary from those of healthy controls, patients with autism had significantly fewer amygdala neurons, especially in the La. Moreover, Bezchlibnyk et al. (2007) and Berretta et al. (2007) reported significant changes in both the somal size, as well as neuron density in the La of subjects with bipolar disorder, relative to healthy controls. These studies imply that in order to illuminate the neural correlates of neuroticism, interindividual differences in the amygdala’s microstructure should be considered, too.

A neuroimaging technique called neurite orientation dispersion and density imaging (NODDI) allows for an in vivo investigation of the microstructural architecture of the human brain (Zhang, Schneider, Wheeler‐Kingshott, & Alexander, 2012). It quantifies neurite morphology based on a multi‐shell high‐angular‐resolution diffusion imaging protocol and offers a novel way to analyze diffusion‐weighted data regarding tissue microstructure in the gray and white matter. NODDI is based on a diffusion model that was successfully validated by histological examinations utilizing staining methods in the gray and white matter of rodents (Jespersen et al., 2010; Jespersen, Leigland, Cornea, & Kroenke, 2012). Zhang et al. (2012) were the first to demonstrate that NODDI is also capable of estimating diffusion markers of neurite density and orientation dispersion by in vivo measurements in humans. A first morphological validation based on a human specimen was conducted by Grussu et al. (2017), who investigated neurite dispersion as a potential marker of multiple sclerosis in postmortem spinal cord samples. The authors reported that neurite density obtained from NODDI significantly matched neurite density, orientation dispersion, and myelin density obtained from histology. This indicates that NODDI metrics are closely reflecting histology. Moreover, current studies have shown that NODDI predicts interindividual differences in cognitive ability (Genç et al., 2018), language processing (Ocklenburg et al., 2018), hemispheric asymmetries (Schmitz et al., 2019), and psychopathology (Nazeri et al., 2017). Aiming to illuminate the neuroanatomical correlates of neuroticism and its facets, the study at hand uses volumetric and NODDI estimates to capture the macrostructural and microstructural architecture of human amygdala nuclei.

## 2 METHODS

### 2.1 Sample size estimation

Since this is the first study investigating the relationship between the macrostructure and microstructure of amygdala nuclei and neuroticism, a literature‐based, a priori sample size estimation could not be executed. Thus, a post hoc test was performed using G‐Power (Faul, Erdfelder, Buchner, & Lang, 2009) to estimate the statistical power. The analysis was based on a linear multiple regression analysis with a small to medium effect size^1^ *ƒ* ^*2*^ = 0.11, *α* = .05, two‐tailed with a sample size of 221 participants. The analysis computed an achieved power of 0.94.

### 2.2 Participants

Two hundred twenty‐one participants between 18 and 35 years of age (*M* = 23.47 years, 115 males) took part in the study. All participants were mentally and neurologically healthy, with no history of psychiatric or neurological disorders, and matched the standard inclusion criteria for MRI examinations. All neurocognitive measures were checked for extreme outliers as defined by Tukey’s fences (Tukey, 1977) approach (observations three interquartile ranges below the first or above the third quartile, respectively), but none were found. Thus, no observations were excluded. The study protocol was approved by the local ethics committee of the Faculty of Psychology at Ruhr University Bochum (vote 165). All participants gave their written informed consent and were treated according to the Declaration of Helsinki.

### 2.3 Acquisition and analysis of neuroticism

To acquire interindividual differences in neuroticism the German version of the Revised NEO Personality Inventory (NEO‐PI‐R) by Ostendorf and Angleitner (2004) was used. The NEO‐PI‐R is a self‐assessment questionnaire that covers the Big Five personality factors (neuroticism, extraversion, openness, conscientiousness, and agreeableness) and their six facets. The questionnaire consists of 240 items. Accordingly, 48 items assess each personality factor. Further, each of the facets consists of eight items. The study at hand focuses on the interindividual differences in neuroticism and the neuroticism facets: Anxiety, angry hostility, depression, self‐conscientiousness, impulsiveness, and vulnerability. Neuroticism is considered to have an internal consistency of *α* = .92 (neuroticism), *α* = .81 (anxiety), *α* = .77 (angry hostility), *α* = .82 (depression), *α* = .69 (self‐conscientiousness), *α* = .65 (impulsiveness), and *α* = .80 (vulnerability; Ostendorf & Angleitner, 2004).

### 2.4 Acquisition of imaging data

All imaging data were acquired at the Bergmannsheil hospital in Bochum, Germany, using a 3T Philips Achieva scanner (Best, The Netherlands) with a 32‐channel head coil.

#### 2.4.1 Anatomical imaging

For segmenting brain scans into gray and white matter sections as well as for the identification of anatomical landmarks a T1‐weighted high‐resolution anatomical image was acquired (MP‐RAGE, TR = 8,179 ms, TE = 3.7 ms, flip angle = 8°, 220 slices, matrix size = 240 × 240, resolution = 1 × 1 × 1 mm). The acquisition time of the anatomical image was about 6 min.

#### 2.4.2 Diffusion‐weighted imaging

To analyze the NODDI coefficients, diffusion‐weighted images were acquired using echo‐planar imaging (TR = 7,652 ms, TE = 87 ms, flip angle = 90°, 60 slices, matrix size = 112 × 112, resolution = 2 × 2 × 2 mm). Diffusion weighting was based on a multi‐shell, high‐angular‐resolution scheme consisting of diffusion‐weighted images for *b* values of 1,000, 1,800, and 2,500 s/mm^2^, respectively, applied along 20, 40, and 60 regularly distributed directions. All diffusion directions within and between shells were generated orthogonal to each other using the MASSIVE toolbox (Froeling, Tax, Vos, Luijten, & Leemans, 2017). Additionally, eight data sets were acquired without diffusion weighting (*b* = 0 s/mm^2^). These unweighted images were used as an anatomical reference for motion correction and computation of NODDI coefficients. The acquisition time of the diffusion‐weighted images was about 18 min.

### 2.5 Analysis of imaging data

#### 2.5.1 Analysis of anatomical data

We used published surface‐based methods in FreeSurfer (http://surfer.nmr.mgh.harvard.edu, version 6.0) to reconstruct the cortical surfaces of the T1‐weighted images. For a detailed description of this procedure, see Dale, Fischl, and Sereno (1999) and Fischl, Sereno, and Dale (1999). The automatic reconstruction steps included skull stripping, segmentation of gray and white matter, as well as reconstruction and inflation of the cortical surface. These processing steps were performed for each participant individually. Subsequently, a slice by slice quality control of the individual segmentation was performed. Inaccuracies in the automatic segmentation were manually corrected if necessary.

**FIGURE 1:**
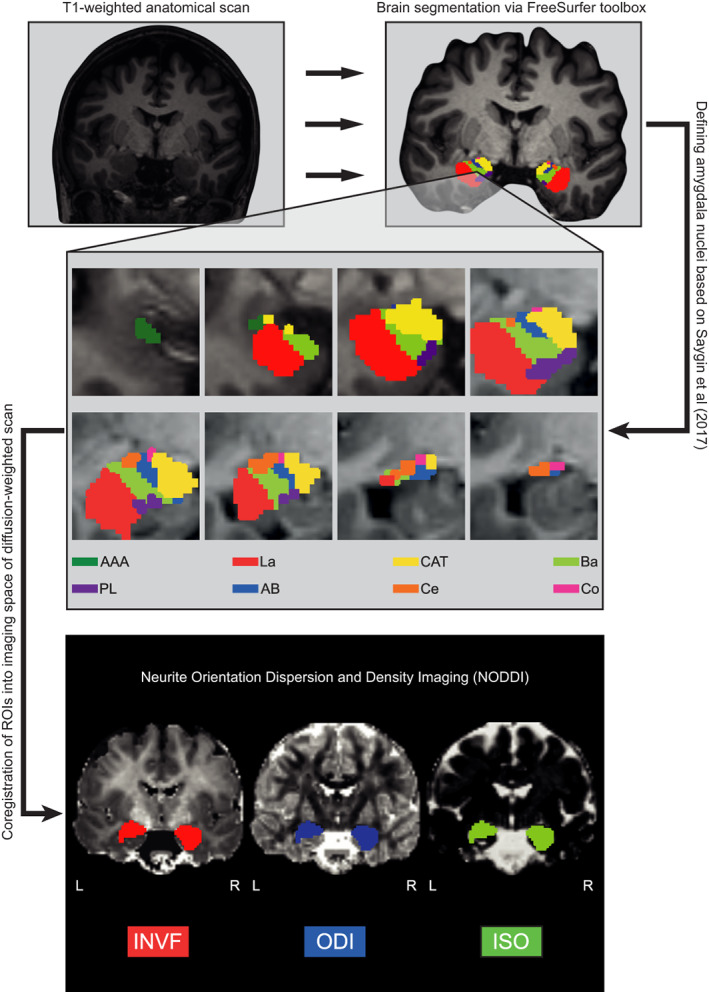
The methodological sequence for the parcellation of the amygdala and the analysis of amygdala nuclei’s microstructure. First, T1‐weighted images were preprocessed using standard FreeSurfer preprocessing steps. Afterward, the amygdala was segmented into eight nuclei per hemisphere according to Saygin et al. (2017). AAA, anterior amygdala area (green); La, lateral nucleus (red); CAT, cortico‐amygdaloid transition area (yellow); Ba, Basal nucleus (light green); PL , paralaminar nucleus (purple); AB, accessory basal nucleus (blue); Ce, central nucleus (orange); Co, cortical nucleus. Second, the volume of each of the amygdala nuclei was computed. Third, the amygdala nuclei were coregistered into the imaging space of the diffusion‐weighted scans. Finally, microstructural NODDI markers were computed: INVF, intra neurite volume fraction (red); ODI, orientation dispersion index (blue); ISO, isotropic diffusion (green). In order to ensure the clarity of the figure, NODDI markers (INVF, ODI, and ISO) were depicted for the total amygdala. However, the respective markers were calculated for each amygdala nucleus

Subcortical gray matter was first segmented into eight areas per hemisphere: thalamus, caudate nucleus, putamen, pallidum, hippocampus, amygdala, and accumbens area (Fischl et al., 2002). Total amygdala volume was derived from this segmentation. Subsequently, we performed an automatic segmentation of the amygdala into nine nuclei per hemisphere: Anterior amygdaloid (AAA) and corticoamygdaloid transition area (CAT), as well as, basal (Ba), lateral (La), accessory basal (AB), central (Ce), cortical (Co), medial (Me), and paralaminar (PL) nuclei (Saygin et al., 2017). The automated parcellation process is based on a probability map and implemented into the most recent version of the FreeSurfer software (FreeSurfer 6.0). Further details of the atlas construction can be found in publications of Saygin et al. (2017), Van Leemput (2009), Van Leemput et al. (2009), and Iglesias et al. (2015). Since the Me could only be segmented in less than half of the 221 participants, subsequent analyses were performed with only eight amygdala nuclei: AAA, CAT, Ba, La, AB, Ce, Co, and PL (Figure 1). Gray matter volume (GMV), estimated by the number of voxels, was calculated for each of the eight amygdala nuclei. Finally, the amygdala nuclei yielded by the parcellation algorithm were linearly transformed into the native space of the diffusion‐weighted images using FreeSurfer’s bbregister function with the ‐‐init‐fsl ‐‐dti option (DF = 6; Greve & Fischl, 2009). The transformed nuclei served as anatomical landmarks from which NODDI coefficients were extracted (Figure 1).

#### 2.5.2 Analysis of diffusion data

FMRIB’s Diffusion Toolbox as implemented in FSL version 5.0.7. was used to preprocess the diffusion‐weighted images. Preprocessing included a correction for eddy currents and head motion, as well as a correction of the gradient direction for each volume using the rotation parameters, which emerged from head motion. NODDI coefficients were computed using the AMICO toolbox (Daducci et al., 2015). The AMICO approach relies on a convex optimization procedure, which converts the nonlinear fitting into a linear optimization problem (Daducci et al., 2015). This allows for a robust estimation of multiple fiber bundles as well as microstructural NODDI indices by dramatically reducing processing time (Sepehrband, Alexander, Kurniawan, Reutens, & Yang, 2016; Tariq, Schneider, Alexander, Wheeler‐Kingshott, & Zhang, 2016). Data analysis with NODDI can be applied to both gray and white matter structures. The NODDI technique is based on a two‐step approach and features a three‐compartment model dividing each voxel into intra‐neurite, extra‐neurite, and cerebrospinal fluid (CSF) environments. First, the diffusion signal obtained by the multi‐shell high‐angular‐resolution imaging protocol is used to determine the rate of free moving water within each voxel (Billiet et al., 2015; Daducci et al., 2015; Jespersen et al., 2010, 2012; Zhang et al., 2012). This ratio is termed isotropic volume fraction (ISO) and reflects the amount of isotropic diffusion with Gaussian properties likely to be found in the CSF of gray and white matter regions. Second, the remaining part of the diffusion signal is divided into the intra‐neurite (INVF) and the extra‐neurite volume fraction (ENVF). By definition, INVF and ENVF complement each other and add up to one (Jespersen et al., 2010, 2012; Zhang et al., 2012). INVF represents the amount of stick‐like or cylindrically symmetric diffusion that is created when the membranes of neurites restrict the diffusion of water molecules. In white matter structures, this kind of diffusion is likely to resemble the proportion of axons. In gray matter regions, such as the amygdala, INVF serves as an indicator of dendrites and axons forming the neuropil. ENVF is based on hindered diffusion within extra‐neurite environments, which is usually precipitated by various types of non‐neuronal cells in white and both neuronal and non‐neuronal cells in gray matter regions (Jespersen et al., 2010, 2012; Zhang et al., 2012). Neurite orientation dispersion (ODI) is a tortuosity measure coupling the intra‐neurite space and the extra‐neurite space resulting in alignment or dispersion of axons in white matter and of axons and dendrites in gray matter (Billiet et al., 2015; Zhang et al., 2012). Examples of ISO, INVF, and ODI coefficient maps from a representative individual are illustrated in Figure 1. As described above, the previously segmented amygdala nuclei were transformed into the native space of the diffusion‐weighted images to compute NODDI coefficients for each of the eight nuclei.

### 2.6 Statistical analysis

Statistical analyses were carried out using Matlab, version 7.14.0.739 (R2012a, The MathWorks Inc., Natick, MA) and SPSS version 25 (SPSS Inc., Chicago, IL). Since the aforementioned neurocognitive measures were normally distributed, as assessed by the Kolmogorov–Smirnov test (*p* > .05), linear parametric methods were used for all analyses. Testing was two‐tailed with an *α*‐level of .05.

#### 2.6.1 Analysis of sex differences

We analyzed our neurocognitive data concerning potential sex differences. To this end, we compared males and females regarding neuroticism, and its facets using two‐sample *t* tests.

#### 2.6.2 Multiple regression analysis

To investigate whether interindividual differences in the macrostructural and microstructural architecture of the amygdala nuclei predict differences in neuroticism or its facets, we computed a total of 28 forced‐entry multiple regression analysis. Here, neuroticism and its facets were treated as dependent variables while the macrostructural or microstructural measures of the amygdala nuclei entered the models as predictors. As previous research indicates a link between age and NODDI (Billiet et al., 2015; Kodiweera, Alexander, Harezlak, McAllister, & Wu, 2016), as well as sex and NODDI (Genc et al., 2018), with our results indicating the same for the amygdala (age: *r* = .321, *p* < .001; sex: see Tables 1, 2, and Tables S1–S3), we added age and sex (male = 0, female = 1) to our regression models. There was no significant correlation between amygdala volume and neurite density (Tables S32 and S33). Finally, all analyses were controlled for multiple comparisons by correcting the *α*‐level using a Bonferroni factor of 28 (*α* = .05/28 = .002).

**TABLE 1:** Sex differences in neuroticism and its facets

	Male		Female		*t*	*p*
	*M*	*SD*	*M*	*SD*		
Neuroticism	77.82	23.35	90.39	20.50	−4.24	.000
Anxiety	13.57	5.45	16.72	5.30	−4.33	.000
Angry hostility	11.43	4.97	13.71	4.59	−3.53	.000
Depression	11.14	6.53	12.79	4.96	−2.13	.034
Self‐consciousness	15.33	5.29	17.47	4.00	−3.41	.001
Impulsiveness	16.28	4.26	17.58	4.58	−2.20	.029
Vulnerability	10.06	4.59	12.11	4.63	−3.31	.001

*Note*: Table 1 depicts sex differences in neuroticism and its facets. Here women tend to achieve significantly higher scores than men. This applies to the main scale as well as to all six facets.

Abbreviations: *M*, mean; *p*, *p* value; *SD*, standard deviation; *t*, *t* value.

**TABLE 2:** Sex differences in amygdala volume

	Male		Female		*t*	*p*
	*M*	*SD*	*M*	*SD*		
Amygdala	3,563.64	355.88	3,286.09	314,13	6.13	.000
AAA	601.82	55.27	549.68	54.58	7.05	.000
AB	126.54	14.53	117.03	12.49	5.20	.000
Ba	988.17	88.92	907.67	80.29	7.04	.000
Ce	101.29	13.22	90.22	12.15	6.46	.000
Co	59.30	7.56	54.16	7.51	5.07	.000
CAT	434.47	39.28	398.78	37.58	6.89	.000
La	1,349.90	123.14	1,221.30	96.05	8.61	.000
PL	112.52	10.97	103.14	9.15	6.87	.000

*Note*: Table 2 depicts sex differences in amygdala volume. Men tend to have significantly higher gray matter volume. This applies to the total amygdala volume as well as to the volume of the nuclei.

Abbreviations: AAA, anterior amygdala area; AB, accessory basal nucleus; Ba, basal nucleus; CAT, Cortico‐amygdaloid transition area; Ce, central nucleus; Co, cortical nucleus; La, lateral nucleus; PL, paralaminar nucleus; *M*, mean; *SD*, standard deviation; *t*, *t* value; *p*, *p* value.

## 3 RESULTS

We present results from a cohort of 221 neurologically and psychologically healthy subjects with a mean age of 23.47 years (*SD* = 3.42), including 115 males and 106 females. We did not observe a significant sex difference with regard to age (*t*[221] = 1.41, *p* = .159). To identify inter‐individual differences in neuroticism, we carried out the German version of the NEO‐PI‐R (Ostendorf & Angleitner, 2004). Our analyses yielded that females (*M* = 90.39, *SD* = 23.35) tend to be significantly more neurotic than males (*M* = 77.82, *SD* = 20.50). This pattern was also evident for the six facets of neuroticism. Across all facets, women tend to have significantly higher scores than men (Table 1).

Regarding amygdala volume, it turned out that women had a significantly lower amygdala volume. This was true for both total amygdala volume and volume of the amygdala nuclei (Table 2).

Subsequently, we analyzed whether interindividual differences in neuroticism and its facets are associated with interindividual differences in the macrostructural and microstructural architecture of the amygdala nuclei: AB, AAA, Ba, Ce, Co, CAT, La, and PL. First, we analyzed the association between neuroticism and GMV of all amygdala nuclei (Figure 1). The respective regression analysis revealed that GMV of none of the amygdala nuclei was significantly associated with individual differences in neuroticism (Tables S4, S8, S12, S16, S20, S24, and S28). Also, at the microstructural level, there was no significant correlation between the NODDI parameters INVF, ODI, and ISO and the subject’s neuroticism score (Tables S5–S7).

**FIGURE 2:**
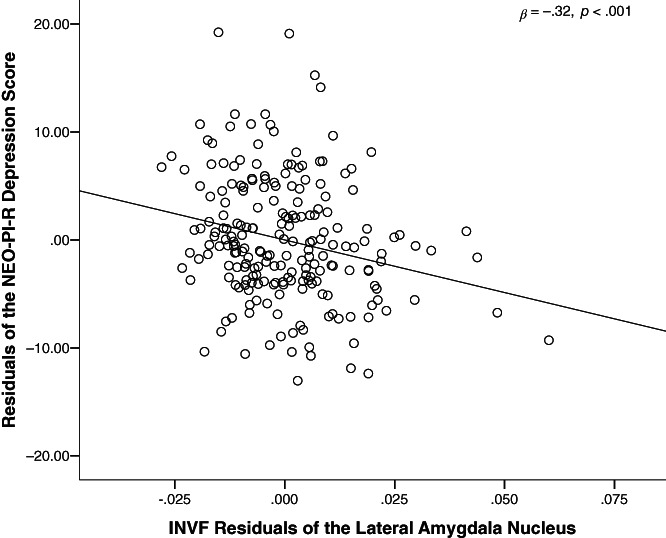
Partial regression plot of La INVF and depression. The scatter plot illustrates the statistically significant relationship between the intra‐neurite volume fraction (INVF) of the lateral amygdala nucleus (La) and depression. The correlation was controlled for sex, age, and INVF of the remaining amygdala nuclei. AAA, anterior amygdala area; AB, accessory basal nucleus; β, standardized beta; Ba, basal nucleus; CAT, cortico‐amygdaloid transition area; Ce, central nucleus; Co, cortical nucleus; La, lateral nucleus; *N*, sample size (*N* = 221); *p*, *p* value; PL, paralaminar nucleus

Since we found no significant association between neuroticism and the macrostructure or microstructure of the amygdala nuclei, we followed the recommendation of Ormel and colleagues and investigated the neural correlates of the neuroticism facets (Ormel, Bastiaansen, et al., 2013; Ormel, Jeronimus, et al., 2013). Here, the neurite density (INVF) of La turned out to be significantly associated with individual differences in the depression subscale of neuroticism. This association was significant above and beyond the effects of sex, age, and INVF of the remaining amygdala nuclei (Table 3). The regression analysis (*F* [10, 211] = 2.26, *p* = .016, *R* ^2^ = .10, *ƒ* ^2^ = .11) revealed that higher INVF in La is associated with a lower level of depression (Table 3 and Figure 2). Due to the previously described sex differences, we conducted an exploratory analysis of this very same regression for both males and females separately. The association pattern between INVF of the LA and the depression facet of neuroticism was comparable to the overall sample in both males (*β* = −.386; *p* = .001) and females (*β* = −.10; *p* = .454). Yet, the regression failed to reach significance in the female sample.

**TABLE 3:** Regression analysis with the depression facet of neuroticism as the outcome variable and INVF of the amygdala nuclei, sex, and age as predictors

	*B*	*SE*	β	*t*	*p*
Constant	34.86	9.92		3.51	.001
AAA	0.45	6.45	.01	0.07	.944
AB	4.45	30.89	.02	0.14	.886
Ba	38.56	39.77	.11	0.97	.333
CAT	0.11	4.61	.00	0.02	.981
Ce	3.55	8.03	.03	0.44	.659
Co	−22.09	11.89	−.14	−1.86	.065
La	−98.11	29.03	−.32	−3.38	.001^a^
PL	7.16	6.28	.08	1.14	.255
Age	0.28	0.94	.02	0.30	.764
Sex	−0.00	0.12	−.00	−0.02	.986

*Note*: *R* ^2^ = .10. Dependent variable: Depression.

Abbreviations: AAA, anterior amygdala area; AB, accessory basal nucleus; β, standardized beta; *B*, unstandardized beta; Ba, basal nucleus; CAT, cortico‐amygdaloid transition area; Ce, central nucleus; Co, cortical nucleus; La, lateral nucleus; *p*, *p* value; PL, paralaminar nucleus; *SE*, standard error; *t*, *t* value.

^a^ The association remains significant after controlling for multiple comparisons by correcting the α‐level using a Bonferroni factor of 28 (*α* = .05/28 = .002).

Interestingly, none of the other regressions led to a significant association between the macrostructure or microstructure of the amygdala nuclei and any of the neuroticism facets (anxiety: Tables S9–S11; angry hostility: Tables S13–S15; depression: Tables S18 and S19; self‐consciousness: Tables S21–S23; impulsivity: Tables S25–S27; vulnerability: Tables S29–S31).

## 4 DISCUSSION

The current study is the first to use NODDI to examine the in vivo macrostructural and microstructural architecture of human amygdala nuclei to illuminate the neural underpinnings of neuroticism. For this purpose, we acquired both high‐resolution brain images and advanced multi‐shell diffusion‐weighted images from 221 healthy individuals. Since the amygdala is not a uniform tissue, but a cluster of nuclei that differ in both structure and function, we automatically segment the amygdala into eight different nuclei. Afterward, we used this segmentation to compute both macrostructural and microstructural markers of each amygdala nucleus. These markers were then linked with the individuals' score in neuroticism and its facets.

Our data indicate no association between the nuclei’s volume and neuroticism or any of its facets. Thus, our results are in line with some of the literature analyzing the relationship between volumetric amygdala differences and neuroticism (Avinun et al., 2019; DeYoung, 2010; Fuentes et al., 2012; Liu et al., 2013; Owens et al., 2019; Wright et al., 2006). However, these studies focused on the amygdala as a whole. In order to gain information about the association between neuroticism and volumetric differences on the level of single nuclei, it once more becomes necessary to evaluate postmortem studies of neuroticism‐related disorders. Here, result patterns are similarly controversial. For instance, Rubinow et al. (2016) report significantly larger volume in La in patients with depression compared to healthy controls, while others like Bowley et al. (2002) could not detect volumetric differences. Others again, even report a volumetric decline in La but not Ba in patients bipolar disorder compared to healthy controls (Berretta et al., 2007). However, it is hard to derive a conclusion from these studies since a major problem of postmortem samples is that they are often restricted to parts of a certain brain area, and thus possible volumetric changes were only partially accounted for (Bowley et al., 2002).

Recent high‐resolution neuroimaging studies examining the relationship between volumetric changes on the level of amygdala nuclei also reveal a rather diverse pattern of association. For instance, Brown et al. (2019) investigated the relationship between volumetric changes on the level of amygdala nuclei and symptom severity in major depression disorder. Their results indicate that volumetric decline was associated with the severity of major depression. Roddy et al. (2021) also demonstrated volumetric differences between patients with major depression disorder and healthy controls. They reported differences in the volume of individual nuclei (e.g., right medial nucleus) as well as differences in left–right volume ratio. A study by Arash Aghamohammadi‐Sereshki et al. (2021) suggests that adverse environmental influences, such as childhood maltreatment, may be related to macrostructural changes in the amygdala and its nuclei. However, the results described for the nuclei did not hold up to the correction for multiple comparisons.

Regarding the microstructural correlates of neuroticism, our study is the first to show that the neurite density (INVF) of La was significantly associated with the individual’s shaping in depression, one of the six neuroticism facets. This effect remained stable after controlling for age and sex. This result partly coincides with previous studies on the microstructural correlates of neuroticism‐related disorders. Although Nazeri et al. (2017) did not show any microstructural differences in the amygdala of patients with either schizophrenia or bipolar disorder compared to healthy controls, they showed that neurite density in the temporal cortex decreased with severity of the disease. Since neuroticism is highly predictive of the diseases examined by Nazeri et al. (2017), one could assume that people with high values in neuroticism, specifically depression, are not only behaviorally but also neurologically at a precursor to these psychiatric disorders. Besides the neuroimaging study of Nazeri et al. (2017), postmortem studies also provide insights that help to interpret our results. Even though these studies almost exclusively focused on the size and number of neurons and glia cells within the amygdala disregarding the dendritic and axonal nature of the respective region of interest (Altshuler et al., 2010; Berretta et al., 2007; Bezchlibnyk et al., 2007; Bowley et al., 2002), they suggest that the observed decline in the neurite density of La is not due to atrophy in neurons, but restricted to changes in the neuropil. An exploratory analysis did not indicate that a separate assessment of the male and female subsamples would have yielded different results. However, contrary to the male sample, the regression analysis carried out for the female sample failed to reach significance. This significance difference might be due to sex related differences in depression, stress response, or emotional processing. Moreover, it might also stem from insufficient statistical power of smaller samples (Mar et al., 2013). Future studies are needed to clarify these matters.

Although the structure–function relationship between INFV in La and increased depression scores cannot be fully explained, our knowledge of the La’s function and the importance of dendritic networks allow for some conclusions. The La is considered to be the sensory relay of the amygdala, evaluating and transmitting incoming information (Duvarci & Pare, 2014; LeDoux et al., 1990; Sigurdsson et al., 2007; Yilmazer‐Hanke, 2012). This information reaches the La bypassing the usual thalamocortical circuits (Orsini & Maren, 2012). Consequently, the amygdala can generate fast, emotionally charged reactions to various stimuli before cortical systems have cognitively categorized the perceptual input (Pape & Pare, 2010). This rapid processing of incoming information is highly adaptive since it allows the organism to quickly react to a threat. However, this is only possible at the expense of accuracy that cortical processing would bring (LeDoux, 1998). During aversive classical conditioning, La neurons undergo fast, NMDA receptor‐dependent synaptic changes (Maren & Quirk, 2004), while La lesions eliminate the expression of conditioned fear (Davis, 2000). Moreover, recordings from La neurons in rodents show the highest spike density under contextual conditions that remind aversive events (Herry et al., 2008; Hobin, Goosens, & Maren, 2003; Maren & Hobin, 2007). Taken together, La neurons can rapidly learn cues that predict aversive events and therefore represent long‐term storage for aversive memories once learned (An, Hong, & Choi, 2012).

INVF reflects the neurite density of a specific brain area and, therefore, its dendritic and axonal density (Zhang et al., 2012). Changes in the dendritic structure of the La could alter its performance significantly. Dendrites constitute most of the surface of a neuron and receive the bulk of synaptic input. While a neuron essentially generates a dichotomous output by producing or not producing an action potential, synaptic integration at the level of dendrites follows different rules (Cazé, Humphries, & Gutkin, 2012, 2013). Since synaptic potentials spread from the site of axodendritic contact to the soma and from there to the axon hillock, dendrites function like leaky electrical cables that nonlinearly filter the thousands of signals that arrive at their membrane surface (Stuart, Spruston, & Häusser, 2016). Past learning events result in selective synaptic strengthening and an increase in the strength of synaptic contacts by input that had been associated with relevant events. As a consequence, somata receive the highest weighted dendritic input encoding the preferred stimulus (Cazé, Jarvis, Foust, & Schultz, 2017). In addition, the spatial position of an input on the dendrite can produce an important stimulus selectivity since synaptic potentials on distal dendrites can be 100‐fold attenuated relative to an input that synapses close to the soma (Häusser, 2001). Overall, dendrites are possibly the most important morphological structure to enable learning‐related signal‐to‐noise filtering in the brain. If subjects with higher depression scores on the NEO‐PI‐R have significantly lower neurite density and, therefore, a lower dendritic density, this could have crucial consequences for these filtering functions of La‐neurons. Although it is impossible to estimate the detailed biophysical processes that would follow, it is likely that complex contextual weighting of sensory input that signals neutral, appetitive, or aversive events could be perturbed. This again might cause harmless sensory information to be misevaluated as threatening and lead to the altered amygdala responsivity that was not only reported in studies on the functional correlates of neuroticism (Brück et al., 2011; Haas et al., 2007; Schweckendiek et al., 2016) but also associated with mood disorders like major depression (Dannlowski et al., 2007; Grotegerd et al., 2014; Mingtian et al., 2012).

Beyond our findings regarding the microstructural architecture of the amygdala nuclei, our work supports the assumption of Ormel and colleagues (Ormel, Bastiaansen, et al., 2013; Ormel, Jeronimus, et al., 2013). None of our regression analyses could reveal a significant neuronal correlate for the overall neuroticism score. However, on the level of facets, a significant association between the microstructural architecture of the amygdala and personality was found.

Although our study makes an important contribution to the field of personality neuroscience, by presenting the first dataset on the microstructural basis of neuroticism, using NODDI, the insights gained are not without limitations. First, our sample was mainly composed of German university students. Thus, it is not representative of the European population in terms of age, educational background, or ethnic composition. Therefore, one must be careful when drawing conclusions about the general population based on our results.

Second, our findings are based on correlations between variables representing measures of the amygdala’s macrostructure and microstructure and the participants’ neuroticism scores and, therefore, do not directly support causal inferences. As an experimental validation of our findings is not feasible, cross‐validation with an independent data set would be desirable. However, to the best of our knowledge, there is currently no available data set, containing diffusion‐weighted neuroimaging data suitable for the derivation of NODDI parameters and comprehensive personality measures that allow for the calculation of the neuroticism facets. We also consider a comparison with other microstructural parameters, such as fractional anisotropy, to be unsuitable, as the relationship between both measures varies even within one brain structure (Friedrich et al., 2020).

Third, our study failed to parcel out all nine amygdala nuclei: AAA, CAT, Ba, La, AB, Ce, Co, Me, and PL (Saygin et al., 2017). Specifically, the Me, a relatively small amygdala nucleus, could only be segmented in less than half of the subjects and was therefore not included in the analysis. Although the automated parcellation procedure developed by Saygin et al. (2017) allows for amygdala segmentation even at lower MRI resolutions and is, therefore, a significant improvement to prior methods, future studies aiming to investigate the amygdala on the level of nuclei, should consider applying ultra‐high field MRI (7‐Tesla or higher) as it drastically improves spatial resolution (Duyn, 2012).

Fourth, regarding the segmentation protocol published by Saygin et al. (2017) it is noteworthy that the protocol was based on a rather small sample of postmortem subjects (*N* = 10). Despite this limited sample, application against two publicly available datasets resulted in accuracies of 59.5–84.0% (Saygin et al., 2017). Therefore, in addition to future use of high‐resolution imaging data, further validation of the segmentation protocol by Saygin et al. (2017) would be desirable.

Fifth, some studies indicate that automated segmentation protocols from various software packages result in a systematic overestimation of amygdala volume compared to manual segmentation (Jayakar et al., 2020; Morey et al., 2009; Schoemaker et al., 2016). According to a study by Alexander et al. (2020), this also applies to the protocol suggested by Saygin et al. (2017). Regarding this point of limitation, it has to be considered that Alexander et al. (2020) compared automatic and manual segmentation in MRI data of higher resolution. However, anatomical landmarks for manual parcellation, like those proposed by Entis et al. (2012) and A. Aghamohammadi‐Sereshki et al. (2018), are hardly transferable to low‐resolution data. Thus, automated parcellation protocols, like the one proposed by Saygin et al. (2017), are currently the most appropriate way to partialize imaging data of low spatial resolution as used in our study. Additionally, as the overestimation caused by automated parcellation seems to be systematic, it mainly affects conclusions about absolute amygdala volume as well as studies comparing absolute volume across several segmentation methods (Alexander et al., 2020).

Sixth, our analysis revealed a significant association between INVF of the lateral amygdala nucleus and the depression score of the NEO‐PI‐R. Even though our study does not allow for conclusions about the functional relationship between La neurite density and depression, we speculate that the association between lower INVF and higher depression scores could be due to changes in the dendritic density. Although a histological validation of Grussu et al. (2017) showed that INVF is indeed representative of neurite density, one must not disregard that it is also partially influenced by orientation dispersion and myelin. To better understand the neurobiological foundation of the INVF, future studies should investigate the relationship between different microstructural measures in both white and gray matter.

Finally, an important factor influencing the robustness of our data is the reproducibility of NODDI. A study by Andica et al. (2020) shows good (intraclass correlation coefficient [ICC] > 0.75) to excellent (ICC > 0.90) within‐session reliability of NODDI markers in both white and gray matter. This was confirmed by Lehmann et al. (2021) who reported 4‐week reliabilities in a similar range (ICC > 0.75). Unfortunately, Lehmann et al. (2021) only investigated white matter. This data suggests that NODDI is inherently reliable. However, we do not know about the reliabilities for NODDI markers in the amygdala specifically, since Andica et al. (2020) only reported reliability measures averaged across all subcortical areas. Future studies should therefore examine both short‐ and long‐term reliabilities of individual cortical and subcortical gray matter regions. In addition, to the best of our knowledge, all previous papers on the reliability of NODDI markers analyzed their data using the original MATLAB tool by Zhang et al. (2012). In our study, however, we used the AMICO toolbox by Daducci et al. (2015). As this toolbox was extensively validated, Lehmann et al. (2021) suggest that previously reported reliability estimates can be considered as benchmarks for NODDI markers obtained using the AMICO toolbox.

In conclusion, our study points toward the significance of the lateral amygdala nucleus regarding neuroticism and related disorders. An essential methodological feature of our study is that, in contrast to previous research, we have examined not only gross structures but also the neural and behavioral compartments. Thus, the insights gained will do justice to both the nature of the amygdala and the conception of neuroticism. Moreover, our results are further evidence that NODDI is a suitable method to study the microstructural foundations of psychological traits and diseases in vivo in humans. Therefore, studies on the microstructural basis of interindividual differences in humans no longer rely solely on small postmortem samples but can also be conducted in large samples of either healthy or diseased subjects.

## CONFLICT OF INTEREST

The authors have declared no conflicts of interest for this article.

## ETHICS STATEMENT

The study protocol was approved by the local ethics committee of the Faculty of Psychology at Ruhr University Bochum (vote 165). All participants gave their written informed consent and were treated according to the Declaration of Helsinki.

## Supporting information


**Appendix S1** Supplementary Information

Supporting Information

## Data Availability

Since the data included in this study are part of an ongoing research project, they are not made openly accessible yet. However, if the manuscript is accepted for publication in Human Brain Mapping, all necessary data will be made publicly available via an OSF link.

## ENDNOTE

^1^
Classification of effect sizes according to Cohen (1992).
