# Supplementary material for: Neurite density imaging in amygdala nuclei reveals interindividual differences in neuroticism
Source: Hum Brain Mapp. 2022 Jan 20;43(6):2051–63. doi: 10.1002/hbm.25775 (PMC8933246; doi:10.1002/hbm.25775)
Supplement: Supplementary file 1 — Appendix S1 Supplementary Information [file HBM-43-2051-s001.pdf]

Sheet	Content
	**T-Test**
**01**	INVF Amygdala + Amygdala nuclei
**02**	ODI Amygdala + Amygdala nuclei
**03**	ISO Amygdala + Amygdala nuclei

	Regression	
	**Dependent Variable**	**Predictors**
**04**	Neuroticism	Sex, Age, AAA_GMV, AB_GMV, Ba_GMV, Ce_GMV, Co_GMV, CAT_GMV, La_GMV, PL_GMV
**05**	Neuroticsm	Sex, Age, AAA_INVF, AB_INVF, Ba_INVF, Ce_INVF, Co_INVF, CAT_INVF, La_INVF, PL_INVF
**06**	Neuroticism	Sex, Age, AAA_ODI, AB_ODI, Ba_ODI, Ce_ODI, Co_ODI, CAT_ODI, La_ODI, PL_ODI
**07**	Neuroticism	Sex, Age, AAA_ISO, AB_ISO, Ba_ISO, Ce_ISO, Co_ISO, CAT_ISO, La_ISO, PL_ISO
**08**	Anxiety	Sex, Age, AAA_GMV, AB_GMV, Ba_GMV, Ce_GMV, Co_GMV, CAT_GMV, La_GMV, PL_GMV
**09**	Anxiety	Sex, Age, AAA_INVF, AB_INVF, Ba_INVF, Ce_INVF, Co_INVF, CAT_INVF, La_INVF, PL_INVF
**10**	Anxiety	Sex, Age, AAA_ODI, AB_ODI, Ba_ODI, Ce_ODI, Co_ODI, CAT_ODI, La_ODI, PL_ODI
**11**	Anxiety	Sex, Age, AAA_ISO, AB_ISO, Ba_ISO, Ce_ISO, Co_ISO, CAT_ISO, La_ISO, PL_ISO
**12**	Angry hostility	Sex, Age, AAA_GMV, AB_GMV, Ba_GMV, Ce_GMV, Co_GMV, CAT_GMV, La_GMV, PL_GMV
**13**	Angry hostility	Sex, Age, AAA_INVF, AB_INVF, Ba_INVF, Ce_INVF, Co_INVF, CAT_INVF, La_INVF, PL_INVF
**14**	Angry hostility	Sex, Age, AAA_ODI, AB_ODI, Ba_ODI, Ce_ODI, Co_ODI, CAT_ODI, La_ODI, PL_ODI
**15**	Angry hostility	Sex, Age, AAA_ISO, AB_ISO, Ba_ISO, Ce_ISO, Co_ISO, CAT_ISO, La_ISO, PL_ISO
**16**	Depression	Sex, Age, AAA_GMV, AB_GMV, Ba_GMV, Ce_GMV, Co_GMV, CAT_GMV, La_GMV, PL_GMV
**17**	Depression	Sex, Age, AAA_INVF, AB_INVF, Ba_INVF, Ce_INVF, Co_INVF, CAT_INVF, La_INVF, PL_INVF
**18**	Depression	Sex, Age, AAA_ODI, AB_ODI, Ba_ODI, Ce_ODI, Co_ODI, CAT_ODI, La_ODI, PL_ODI
**19**	Depression	Sex, Age, AAA_ISO, AB_ISO, Ba_ISO, Ce_ISO, Co_ISO, CAT_ISO, La_ISO, PL_ISO
**20**	Self-consciousness	Sex, Age, AAA_GMV, AB_GMV, Ba_GMV, Ce_GMV, Co_GMV, CAT_GMV, La_GMV, PL_GMV
**21**	Self-consciousness	Sex, Age, AAA_INVF, AB_INVF, Ba_INVF, Ce_INVF, Co_INVF, CAT_INVF, La_INVF, PL_INVF
**22**	Self-consciousness	Sex, Age, AAA_ODI, AB_ODI, Ba_ODI, Ce_ODI, Co_ODI, CAT_ODI, La_ODI, PL_ODI
**23**	Self-consciousness	Sex, Age, AAA_ISO, AB_ISO, Ba_ISO, Ce_ISO, Co_ISO, CAT_ISO, La_ISO, PL_ISO
**24**	Impulsivity	Sex, Age, AAA_GMV, AB_GMV, Ba_GMV, Ce_GMV, Co_GMV, CAT_GMV, La_GMV, PL_GMV
**25**	Impulsivity	Sex, Age, AAA_INVF, AB_INVF, Ba_INVF, Ce_INVF, Co_INVF, CAT_INVF, La_INVF, PL_INVF
**26**	Impulsivity	Sex, Age, AAA_ODI, AB_ODI, Ba_ODI, Ce_ODI, Co_ODI, CAT_ODI, La_ODI, PL_ODI
**27**	Impulsivity	Sex, Age, AAA_ISO, AB_ISO, Ba_ISO, Ce_ISO, Co_ISO, CAT_ISO, La_ISO, PL_ISO
**28**	Vulnerability	Sex, Age, AAA_GMV, AB_GMV, Ba_GMV, Ce_GMV, Co_GMV, CAT_GMV, La_GMV, PL_GMV
**29**	Vulnerability	Sex, Age, AAA_INVF, AB_INVF, Ba_INVF, Ce_INVF, Co_INVF, CAT_INVF, La_INVF, PL_INVF
**30**	Vulnerability	Sex, Age, AAA_ODI, AB_ODI, Ba_ODI, Ce_ODI, Co_ODI, CAT_ODI, La_ODI, PL_ODI
**31**	Vulnerability	Sex, Age, AAA_ISO, AB_ISO, Ba_ISO, Ce_ISO, Co_ISO, CAT_ISO, La_ISO, PL_ISO

	Correlation
**32**	GMV + INVF (Age, Sex)
**33**	GMV + ODI (Age, Sex)

Key		
**Neuroimaging measures**		
GMV	Gray matter volume	Macrostructural measure
INVF	Intra neurite volume fraction	Microstructural measure
ODI	Orientation dispersion index	
ISO	Isotropic diffusion	
**Amygdala Nuclei according to Saygin et al. 2017**		
AAA	Anterior amygdala area	
AB	Accessory basal nucleus	
Ba	Basal nucleus	
Ce	Central nucleus	
Co	Cortical nucleus	
CAT	Cortico-amygdaloid transition area	
La	Lateral nucleus	
PL	Paralaminar nucleus	

Group Statistics					
Sex		N	Mean	SD	SE of Mean
AMYG_INVF	**male**	115,000	0,339	0,014	0,001
	**female**	106,000	0,324	0,014	0,001
AAA_INVF	**male**	115,000	0,317	0,020	0,002
	**female**	106,000	0,300	0,018	0,002
AB_INVF	**male**	115,000	0,264	0,059	0,005
	**female**	105,000	0,237	0,066	0,006
Ba_INVF	**male**	115,000	0,330	0,016	0,001
	**female**	106,000	0,317	0,013	0,001
Ce_INVF	**male**	115,000	0,338	0,046	0,004
	**female**	106,000	0,302	0,059	0,006
Co_INVF	**male**	115,000	0,341	0,040	0,004
	**female**	106,000	0,324	0,033	0,003
CAT_INVF	**male**	112,000	0,311	0,091	0,009
	**female**	101,000	0,287	0,083	0,008
La_INVF	**male**	115,000	0,344	0,016	0,001
	**female**	106,000	0,326	0,018	0,002
PL_INVF	**male**	114,000	0,290	0,069	0,006
	**female**	106,000	0,285	0,060	0,006
N = sample size, SD = standard deviation, SE = standard error					

Independent Samples Test										
		Levene's Tests for		t-test for Equality of Means						
		F	p	t	df	p	Mean Difference	SE of Mean	95% Confidence Lower	Upper
AMYG_INVF	**Equal variences assumed**	0,165	0,685	8,242	219,000	0,000	0,015	0,002	0,012	0,019
	**Equal variences not assumed**			8,244	217,670	0,000	0,015	0,002	0,012	0,019
AAA_INVF	**Equal variences assumed**	1,414	0,236	6,717	219,000	0,000	0,017	0,003	0,012	0,022
	**Equal variences not assumed**			6,747	218,844	0,000	0,017	0,003	0,012	0,022
AB_INVF	**Equal variences assumed**	7,525	0,007	3,202	218,000	0,002	0,027	0,008	0,010	0,044
	**Equal variences not assumed**			3,185	208,972	0,002	0,027	0,008	0,010	0,044
Ba_INVF	**Equal variences assumed**	2,217	0,138	6,325	219,000	0,000	0,013	0,002	0,009	0,016
	**Equal variences not assumed**			6,369	217,299	0,000	0,013	0,002	0,009	0,016
Ce_INVF	**Equal variences assumed**	3,142	0,078	5,051	219,000	0,000	0,036	0,007	0,022	0,049
	**Equal variences not assumed**			5,001	198,141	0,000	0,036	0,007	0,022	0,050
Co_INVF	**Equal variences assumed**	0,651	0,421	3,437	219,000	0,001	0,017	0,005	0,007	0,027
	**Equal variences not assumed**			3,461	217,111	0,001	0,017	0,005	0,007	0,027
CAT_INVF	**Equal variences assumed**	0,007	0,933	2,056	211,000	0,041	0,025	0,012	0,001	0,048
	**Equal variences not assumed**			2,065	210,962	0,040	0,025	0,012	0,001	0,048
La_INVF	**Equal variences assumed**	2,132	0,146	7,863	219,000	0,000	0,018	0,002	0,013	0,022
	**Equal variences not assumed**			7,821	209,742	0,000	0,018	0,002	0,013	0,022
PL_INVF	**Equal variences assumed**	2,071	0,152	0,616	218,000	0,538	0,005	0,009	-0,012	0,023
	**Equal variences not assumed**			0,619	217,174	0,536	0,005	0,009	-0,012	0,023
F = F-value, p = p-value, t = t-value, df = degrees of freedom, SE = standard error										

Group Statistics					
Sex		N	Mean	SD	SE of Mean
AMYG_ODI	**male**	115,000	0,436	0,024	0,002
	**female**	106,000	0,433	0,024	0,002
AAA_ODI	**male**	115,000	0,443	0,029	0,003
	**female**	106,000	0,445	0,033	0,003
AB_ODI	**male**	115,000	0,398	0,102	0,010
	**female**	105,000	0,381	0,125	0,012
Ba_ODI	**male**	115,000	0,491	0,032	0,003
	**female**	106,000	0,491	0,031	0,003
Ce_ODI	**male**	115,000	0,325	0,052	0,005
	**female**	106,000	0,313	0,080	0,008
Co_ODI	**male**	115,000	0,395	0,036	0,003
	**female**	106,000	0,388	0,034	0,003
CAT_ODI	**male**	112,000	0,155	0,055	0,005
	**female**	101,000	0,155	0,062	0,006
La_ODI	**male**	115,000	0,443	0,045	0,004
	**female**	106,000	0,441	0,039	0,004
PL_ODI	**male**	114,000	0,453	0,127	0,012
	**female**	106,000	0,456	0,111	0,011

N = sample size, SD = standard deviation, SE = standard error

Independent Samples Test										
		Levene's Tests for		t-test for Equality of Means						
							Mean	SE of	95% Confidence	
		F	p	t	df	p	Difference	Mean	Lower	Upper
AMYG_ODI	**Equal variences assumed**	0,053	0,818	0,981	219,000	0,328	0,003	0,003	-0,003	0,010
	**Equal variences not assumed**			0,981	217,971	0,328	0,003	0,003	-0,003	0,010
AAA_ODI	**Equal variences assumed**	4,613	0,033	-0,509	219,000	0,611	-0,002	0,004	-0,010	0,006
	**Equal variences not assumed**			-0,506	208,870	0,613	-0,002	0,004	-0,010	0,006
AB_ODI	**Equal variences assumed**	5,760	0,017	1,138	218,000	0,256	0,018	0,015	-0,013	0,048
	**Equal variences not assumed**			1,128	201,057	0,261	0,018	0,016	-0,013	0,048
Ba_ODI	**Equal variences assumed**	0,067	0,797	0,153	219,000	0,879	0,001	0,004	-0,008	0,009
	**Equal variences not assumed**			0,153	218,741	0,878	0,001	0,004	-0,008	0,009
Ce_ODI	**Equal variences assumed**	8,697	0,004	1,245	219,000	0,215	0,011	0,009	-0,007	0,029
	**Equal variences not assumed**			1,224	176,975	0,223	0,011	0,009	-0,007	0,029
Co_ODI	**Equal variences assumed**	0,012	0,914	1,495	219,000	0,136	0,007	0,005	-0,002	0,016
	**Equal variences not assumed**			1,498	218,861	0,136	0,007	0,005	-0,002	0,016
CAT_ODI	**Equal variences assumed**	1,003	0,318	0,012	211,000	0,990	0,000	0,008	-0,016	0,016
	**Equal variences not assumed**			0,012	201,482	0,990	0,000	0,008	-0,016	0,016
La_ODI	**Equal variences assumed**	1,754	0,187	0,445	219,000	0,657	0,003	0,006	-0,009	0,014
	**Equal variences not assumed**			0,447	218,099	0,655	0,003	0,006	-0,009	0,014
PL_ODI	**Equal variences assumed**	2,501	0,115	-0,171	218,000	0,864	-0,003	0,016	-0,034	0,029
	**Equal variences not assumed**			-0,172	217,180	0,864	-0,003	0,016	-0,034	0,029

F = F-value, p = p-value, t = t-value, df = degrees of freedom, SE = standard error

Group Statistics					
Sex		N	Mean	SD	SE of Mean
AMYG_ISO	male	115,000	0,383	0,055	0,005
	female	106,000	0,382	0,054	0,005
AAA_ISO	male	115,000	0,363	0,095	0,009
	female	106,000	0,375	0,090	0,009
AB_ISO	male	46,000	0,147	0,118	0,017
	female	41,000	0,131	0,105	0,016
Ba_ISO	male	115,000	0,359	0,096	0,009
	female	106,000	0,384	0,089	0,009
Ce_ISO	male	94,000	0,211	0,161	0,017
	female	79,000	0,214	0,145	0,016
Co_ISO	male	115,000	0,338	0,105	0,010
	female	106,000	0,348	0,079	0,008
CAT_ISO	male	77,000	0,160	0,143	0,016
	female	47,000	0,145	0,118	0,017
La_ISO	male	115,000	0,391	0,092	0,009
	female	106,000	0,372	0,084	0,008
PL_ISO	male	31,000	0,079	0,071	0,013
	female	20,000	0,096	0,086	0,019

N = sample size, SD = standard deviation, SE = standard error

Independent Samples Test										
		Levene's Tests for		t-test for Equality of Means						
		F	p	t	df	p	Mean Difference	SE of Mean	95% Confidence Lower	Upper
AMYG_ODI	Equal variences assumed	0,059	0,809	0,107	219,000	0,915	0,001	0,007	-0,014	0,015
	Equal variences not assumed			0,107	217,994	0,915	0,001	0,007	-0,014	0,015
AAA_ODI	Equal variences assumed	0,306	0,580	-0,942	219,000	0,347	-0,012	0,013	-0,036	0,013
	Equal variences not assumed			-0,944	218,863	0,346	-0,012	0,012	-0,036	0,013
AB_ODI	Equal variences assumed	0,724	0,397	0,691	85,000	0,492	0,017	0,024	-0,031	0,065
	Equal variences not assumed			0,695	85,000	0,489	0,017	0,024	-0,031	0,064
Ba_ODI	Equal variences assumed	1,199	0,275	-1,951	219,000	0,052	-0,024	0,012	-0,049	0,000
	Equal variences not assumed			-1,957	218,995	0,052	-0,024	0,012	-0,049	0,000
Ce_ODI	Equal variences assumed	1,618	0,205	-0,122	171,000	0,903	-0,003	0,023	-0,049	0,043
	Equal variences not assumed			-0,123	170,052	0,902	-0,003	0,023	-0,049	0,043
Co_ODI	Equal variences assumed	4,622	0,033	-0,804	219,000	0,422	-0,010	0,013	-0,035	0,015
	Equal variences not assumed			-0,813	210,960	0,417	-0,010	0,012	-0,035	0,014
CAT_ODI	Equal variences assumed	0,522	0,471	0,610	122,000	0,543	0,015	0,025	-0,034	0,064
	Equal variences not assumed			0,639	111,232	0,524	0,015	0,024	-0,032	0,062
La_ODI	Equal variences assumed	1,013	0,315	1,533	219,000	0,127	0,018	0,012	-0,005	0,042
	Equal variences not assumed			1,539	218,963	0,125	0,018	0,012	-0,005	0,042
PL_ODI	Equal variences assumed	1,619	0,209	-0,775	49,000	0,442	-0,017	0,022	-0,062	0,027
	Equal variences not assumed			-0,742	34,972	0,463	-0,017	0,023	-0,064	0,030

F = F-value, p = p-value, t = t-value, df = degrees of freedom, SE = standard error

Model Summary					
**Model**	**R**	**R Square**	**Adjusted R Square**	**SE of the Estimate**	f^2
**1**	0,319[a]	0,102	0,059	22,176	0,114
a. Predictors: (Constant), Sex, Age, AAA_GMV, AB_GMV, Ba_GMV, Ce_GMV, Co_GMV, CAT_GMV, La_GMV, PL_GMV					
R = R value; SE = standard error; $f2$ = Cohen's $f2$					

a. Dependent Variable: NEO_NEU						
**Model**		**Sum of Squares**	**df**	**Mean Square**	**F**	**p**
**1**	**Regression**	11715,160	10,000	1171,516	2,382	,011[b]
	**Residual**	103273,610	210,000	491,779		
	**Total**	114988,769	220,000			
a. Dependent Variable: Neuroticism						
b. Predictors: (Constant), Sex, Age, AAA_GMV, AB_GMV, Ba_GMV, Ce_GMV, Co_GMV, CAT_GMV, La_GMV, PL_GMV						
df = degrees of freedom; F = F value; p = p value						

Coefficients[a]						
**Model**		**Unstandardized Coefficients**		**Standardized Coefficients**	**t**	**p**
		**B**	**SE**	**β**		
**1**	**(Constant)**	69,730	22,904		3,044	0,003
	**Sex**	14,574	3,600	0,319	4,048	0,000
	**Age**	-0,693	0,449	-0,104	-1,545	0,124
	**AAA_GMV**	-0,084	0,144	-0,224	-0,583	0,560
	**AB_GMV**	0,041	0,186	0,026	0,222	0,825
	**Ba_GMV**	-0,052	0,110	-0,212	-0,468	0,640
	**Ce_GMV**	0,072	0,172	0,044	0,419	0,676
	**Co_GMV**	0,264	0,448	0,092	0,589	0,557
	**CAT_GMV**	0,081	0,098	0,150	0,826	0,409
	**La_GMV**	0,020	0,028	0,112	0,714	0,476
	**PL_GMV**	0,319	0,479	0,156	0,666	0,506
a. Dependent Variable: Neuroticism						
B = unstandardized beta; SE = standard error; β = standardized beta; t = t value; p = p value						

Model Summary					
**Model**	**R**	**R^2**	**Adjusted R^2**	**SE of the Estimate**	f^2
**1**	0,382[a]	0,146	0,104	21,646	0,171
a. Predictors: (Constant), Sex, Age, AAA_INVF, AB_INVF, Ba_INVF, Ce_INVF, Co_INVF, CAT_INVF, La_INVF, PL_INVF					
R = R value; SE = standard error; $f2$ = Cohen's $f2$					

ANOVA[a]						
**Model**		**Sum of Squares**	**df**	**Mean Square**	**F**	**p**
**1**	**Regression**	16106,810	10,000	1610,681	3,438	0,000[b]
	**Residual**	94177,873	201,000	468,547		
	**Total**	110284,683	211,000			
a. Dependent Variable: Neuroticism						
b. Predictors: (Constant), Sex, Age, AAA_INVF, AB_INVF, Ba_INVF, Ce_INVF, Co_INVF, CAT_INVF, La_INVF, PL_INVF						
df = degrees of freedom; F = F value; p = p value						

Coefficients[a]						
**Model**		**Unstandardized Coefficients**		**Standardized Coefficients**	**t**	**p**
		**B**	**SE**	**β**		
**1**	**(Constant)**	184,333	37,661		4,895	0,000
	**Sex**	6,979	3,581	0,153	1,949	0,053
	**Age**	-0,237	0,471	-0,035	-0,503	0,616
	**AAA_INVF**	-33,236	117,258	-0,030	-0,283	0,777
	**AB_INVF**	15,240	24,475	0,043	0,623	0,534
	**Ba_INVF**	68,236	150,957	0,048	0,452	0,652
	**Ce_INVF**	-2,520	30,482	-0,006	-0,083	0,934
	**Co_INVF**	-74,233	45,124	-0,122	-1,645	0,102
	**CAT_INVF**	11,732	17,571	0,045	0,668	0,505
	**La_INVF**	-294,052	110,197	-0,243	-2,668	0,008
	**PL_INVF**	22,759	23,817	0,064	0,956	0,340
a. Dependent Variable: Neuroticism;						
B = unstandardized beta; SE = standard error; β = standardized beta; t = t value; p = p value						

Model Summary					
**Model**	**R**	**R Square**	**Adjusted R Square**	**SE of the Estimate**	f^2
**1**	0,372[a]	0,138	0,095	21,747	0,160
a. Predictors: (Constant), Sex, Age, AAA_ODI, AB_ODI, Ba_ODI, Ce_ODI, Co_ODI, CAT_ODI, La_ODI, PL_ODI					
R = R value; SE = standard error; $f2$ = Cohen's $f2$					

ANOVA[a]						
**Model**		**Sum of Squares**	**df**	**Mean Square**	**F**	**p**
1	**Regression**	15226,300	10,000	1522,630	3,220	0,001[b]
	**Residual**	95058,383	201,000	472,927		
	**Total**	110284,683	211,000			
a. Dependent Variable: Neuroticism						
b. Predictors: (Constant), Sex, Age, AAA_ODI, AB_ODI, Ba_ODI, Ce_ODI, Co_ODI, CAT_ODI, La_ODI, PL_ODI						
df = degrees of freedom; F = F value; p = p value						

Coefficients[a]						
**Model**		**Unstandardized Coefficients**		**Standardized Coefficients**	**t**	**p**
		**B**	**SE**	**β**		
1	**(Constant)**	116,143	31,838		3,648	0,000
	**Sex**	11,602	3,069	0,254	3,780	0,000
	**Age**	-0,672	0,450	-0,101	-1,492	0,137
	**AAA_ODI**	-32,228	64,119	-0,044	-0,503	0,616
	**AB_ODI**	10,800	14,441	0,054	0,748	0,455
	**Ba_ODI**	89,324	63,827	0,122	1,399	0,163
	**Ce_ODI**	-4,745	24,159	-0,014	-0,196	0,844
	**Co_ODI**	-69,603	53,185	-0,106	-1,309	0,192
	**CAT_ODI**	48,107	26,459	0,122	1,818	0,071
	**La_ODI**	-100,335	44,004	-0,184	-2,280	0,024
	**PL_ODI**	21,681	12,939	0,113	1,676	0,095
a. Dependent Variable: Neuroticism						
B = unstandardized beta; SE = standard error; β = standardized beta; t = t value; p = p value						

Model Summary					
**Model**	**R**	**R Square**	**Adjusted R Square**	**SE of the Estimate**	f^2
1	0,378[a]	0,143	-1,081	32,979	0,167
a. Predictors: (Constant), Sex, Age, AAA_ISO, AB_ISO, Ba_ISO, Ce_ISO, Co_ISO, CAT_ISO, La_ISO, PL_ISO					
R = R value; SE = standard error; $f2$ = Cohen's $f2$					

ANOVA[a]						
**Model**		**Sum of Squares**	**df**	**Mean Square**	**F**	**p**
**1**	**Regression**	1272,039	10,000	127,204	0,117	0,998[b]
	**Residual**	7613,457	7,000	1087,637		
	**Total**	8885,496	17,000			
a. Dependent Variable: Neuroticism						
b. Predictors: (Constant), Sex, Age, AAA_ISO, AB_ISO, Ba_ISO, Ce_ISO, Co_ISO, CAT_ISO, La_ISO, PL_ISO						
df = degrees of freedom; F = F value; p = p value						

Coefficients[a]						
**Model**		**Unstandardized Coefficients**		**Standardized Coefficients**	**t**	**p**
		**B**	**SE**	**β**		
**1**	**(Constant)**	118,412	81,312		1,456	0,189
	**Sex**	12,710	16,637	0,278	0,764	0,470
	**Age**	-0,462	2,410	-0,069	-0,192	0,853
	**AAA_ISO**	7,202	102,486	0,029	0,070	0,946
	**AB_ISO**	-0,431	84,429	-0,002	-0,005	0,996
	**Ba_ISO**	-24,416	101,821	-0,100	-0,240	0,817
	**Ce_ISO**	-7,108	60,211	-0,048	-0,118	0,909
	**Co_ISO**	-10,089	95,006	-0,041	-0,106	0,918
	**CAT_ISO**	9,200	62,052	0,054	0,148	0,886
	**La_ISO**	-45,198	94,764	-0,175	-0,477	0,648
	**PL_ISO**	-29,749	121,712	-0,100	-0,244	0,814
a. Dependent Variable: Neuroticism						
B = unstandardized beta; SE = standard error; β = standardized beta; t = t value; p = p value						

Model Summary					
**Model**	**R**	**R Square**	**Adjusted R Square**	**SE of the Estimate**	f^2
**1,000**	0,353[a]	0,124	0,083	5,366	0,142
a. Predictors: (Constant), Sex, Age, AAA_GMV, AB_GMV, Ba_GMV, Ce_GMV, Co_GMV, CAT_GMV, La_GMV, PL_GMV					
R = R value; SE = standard error; $f2$ = Cohen's $f2$					

ANOVA[a]						
**Model**		**Sum of Squares**	**df**	**Mean Square**	**F**	**p**
**1,000**	**Regression**	857,981	10,000	85,798	2,980	0,002[b]
	**Residual**	6046,553	210,000	28,793		
	**Total**	6904,534	220,000			
a. Dependent Variable: Anxiety						
b. Predictors: (Constant) Sex, Age, AAA_GMV, AB_GMV, Ba_GMV, Ce_GMV, Co_GMV, CAT_GMV, La_GMV, PL_GMV						
df = degrees of freedom; F = F value; p = p value						

Coefficients[a]						
**Model**		**Unstandardized Coefficients**		**Standardized Coefficients**	**t**	**p**
		**B**	**SE**	**β**		
**1,000**	**(Constant)**	12,163	5,542		2,195	0,029
	**Sex**	3,782	0,871	0,338	4,342	0,000
	**Age**	-0,190	0,109	-0,116	-1,746	0,082
	**AAA_GMV**	0,002	0,035	0,025	0,066	0,948
	**AB_GMV**	-0,051	0,045	-0,131	-1,136	0,257
	**Ba_GMV**	-0,026	0,027	-0,443	-0,992	0,323
	**Ce_GMV**	-0,009	0,042	-0,022	-0,212	0,832
	**Co_GMV**	0,042	0,108	0,059	0,385	0,701
	**CAT_GMV**	0,011	0,024	0,086	0,477	0,634
	**La_GMV**	0,011	0,007	0,260	1,678	0,095
	**PL_GMV**	0,136	0,116	0,271	1,176	0,241
a. Dependent Variable: Anxiety						
B = unstandardized beta; SE = standard error; β = standardized beta; t = t value; p = p value						

Model Summary					
Model	R	R Square	Adjusted R Square	SE of the Estimate	f^2
1	0,357[a]	0,127	0,084	5,362	0,145
a. Predictors: (Constant), Sex, Age, AAA_INVF, AB_INVF, Ba_INVF, Ce_INVF, Co_INVF, CAT_INVF, La_INVF, PL_INVF					
R = R value; SE = standard error; $f2$ = Cohen's $f2$					

ANOVA[a]						
Model		Sum of Squares	df	Mean Square	F	p
1	Regression	842,098	10,000	84,210	2,928	0,002[b]
	Residual	5779,978	201,000	28,756		
	Total	6622,076	211,000			
a. Dependent Variable: Anxiety						
b. Predictors: (Constant), Sex, Age, AAA_INVF, AB_INVF, Ba_INVF, Ce_INVF, Co_INVF, CAT_INVF, La_INVF, PL_INVF						
df = degrees of freedom; F = F value; p = p value						

Coefficients[a]						
Model		Unstandardized Coefficients		Standardized Coefficients	t	p
		B	SE	β		
1	(Constant)	36,395	9,330		3,901	0,000
	Sex	2,021	0,887	0,181	2,278	0,024
	Age	-0,076	0,117	-0,046	-0,653	0,515
	AAA_INVF	20,879	29,049	0,077	0,719	0,473
	AB_INVF	4,168	6,063	0,048	0,687	0,493
	Ba_INVF	-15,631	37,397	-0,045	-0,418	0,676
	Ce_INVF	-9,402	7,552	-0,093	-1,245	0,215
	Co_INVF	-13,612	11,179	-0,091	-1,218	0,225
	CAT_INVF	2,989	4,353	0,047	0,687	0,493
	La_INVF	-48,261	27,300	-0,162	-1,768	0,079
	PL_INVF	-0,360	5,900	-0,004	-0,061	0,951
a. Dependent Variable: Anxiety						
B = unstandardized beta; SE = standard error; β = standardized beta; t = t value; p = p value						

Model Summary					
**Model**	**R**	**R Square**	**Adjusted R Square**	**SE of the Estimate**	f^2
**1**	0,338[a]	0,114	0,070	5,401	0,129
a. Predictors: (Constant), Sex, Age, AAA_ODI, AB_ODI, Ba_ODI, Ce_ODI, Co_ODI, CAT_ODI, La_ODI, PL_ODI					
R = R value; SE = standard error; $f2$ = Cohen's $f2$					

ANOVA[a]						
**Model**		**Sum of Squares**	**df**	**Mean Square**	**F**	**p**
1	**Regression**	757,827	10,000	75,783	2,597	0,006[b]
	**Residual**	5864,249	201,000	29,175		
	**Total**	6622,076	211,000			
a. Dependent Variable: Anxiety						
b. Predictors: (Constant), Sex, Age, AAA_ODI, AB_ODI, Ba_ODI, Ce_ODI, Co_ODI, CAT_ODI, La_ODI, PL_ODI						
df = degrees of freedom; F = F value; p = p value						

Coefficients[a]						
**Model**		**Unstandardized Coefficients**		**Standardized Coefficients**	**t**	**p**
		**B**	**SE**	**β**		
1	**(Constant)**	24,175	7,908		3,057	0,003
	**Sex**	2,925	0,762	0,261	3,837	0,000
	**Age**	-0,151	0,112	-0,092	-1,347	0,179
	**AAA_ODI**	-6,220	15,926	-0,035	-0,391	0,697
	**AB_ODI**	2,984	3,587	0,061	0,832	0,406
	**Ba_ODI**	18,542	15,853	0,103	1,170	0,244
	**Ce_ODI**	-3,689	6,000	-0,044	-0,615	0,539
	**Co_ODI**	-15,196	13,210	-0,094	-1,150	0,251
	**CAT_ODI**	7,314	6,572	0,076	1,113	0,267
	**La_ODI**	-19,698	10,929	-0,148	-1,802	0,073
	**PL_ODI**	0,534	3,214	0,011	0,166	0,868
a. Dependent Variable: Anxiety						
B = unstandardized beta; SE = standard error; β = standardized beta; t = t value; p = p value						

Model Summary					
**Model**	**R**	**R Square**	**Adjusted R Square**	**SE of the Estimate**	f^2
**1**	0,392[a]	0,154	-1,055	8,032	0,182

a. Predictors: (Constant), Sex, Age, AAA_ISO, AB_ISO, Ba_ISO, Ce_ISO, Co_ISO, CAT_ISO, La_ISO, PL_ISO

R = R value; SE = standard error; $f2$ = Cohen's $f2$

ANOVA[a]						
**Model**		**Sum of Squares**	**df**	**Mean Square**	**F**	**p**
**1**	**Regression**	81,965	10,000	8,197	0,127	0,998[b]
	**Residual**	451,567	7,000	64,510		
	**Total**	533,532	17,000			

a. Dependent Variable: Anxiety

b. Predictors: (Constant), Sex, Age, AAA_ISO, AB_ISO, Ba_ISO, Ce_ISO, Co_ISO, CAT_ISO, La_ISO, PL_ISO

df = degrees of freedom; F = F value; p = p value

Coefficients[a]						
**Model**		**Unstandardized Coefficients**		**Standardized Coefficients**	**t**	**p**
		**B**	**SE**	**β**		
**1**	**(Constant)**	23,538	19,803		1,189	0,273
	**Sex**	3,260	4,052	0,291	0,805	0,447
	**Age**	-0,107	0,587	-0,065	-0,182	0,861
	**AAA_ISO**	3,823	24,959	0,063	0,153	0,883
	**AB_ISO**	-1,938	20,562	-0,039	-0,094	0,928
	**Ba_ISO**	-11,759	24,797	-0,196	-0,474	0,650
	**Ce_ISO**	-2,662	14,664	-0,073	-0,182	0,861
	**Co_ISO**	-0,867	23,138	-0,014	-0,037	0,971
	**CAT_ISO**	1,603	15,112	0,038	0,106	0,919
	**La_ISO**	-8,422	23,079	-0,133	-0,365	0,726
	**PL_ISO**	-5,397	29,642	-0,074	-0,182	0,861

a. Dependent Variable: Anxiety

B = unstandardized beta; SE = standard error; β = standardized beta; t = t value; p = p value

Model Summary					
Model	R	R Square	Adjusted R Square	SE of the Estimate	f^2
1	0,302[a]	0,091	0,048	4,793	0,100
a. Predictors: (Constant), Sex, Age, AAA_GMV, AB_GMV, Ba_GMV, Ce_GMV, Co_GMV, CAT_GMV, La_GMV, PL_GMV					
R = R value; SE = standard error; $f2$ = Cohen's $f2$					

ANOVA[a]						
Model		Sum of Squares	df	Mean Square	F	p
1	Regression	483,783	10,000	48,378	2,106	0,025[b]
	Residual	4823,330	210,000	22,968		
	Total	5307,113	220,000			
a. Dependent Variable: Angry hostility						
b. Predictors: (Constant), Sex, Age, AAA_GMV, AB_GMV, Ba_GMV, Ce_GMV, Co_GMV, CAT_GMV, La_GMV, PL_GMV						
df = degrees of freedom; F = F value; p = p value						

Coefficients[a]						
Model		Unstandardized Coefficients		Standardized Coefficients	t	p
		B	SE	β		
1	(Constant)	13,231	4,950		2,673	0,008
	Sex	2,408	0,778	0,245	3,095	0,002
	Age	-0,160	0,097	-0,112	-1,652	0,100
	AAA_GMV	-0,048	0,031	-0,591	-1,532	0,127
	AB_GMV	0,005	0,040	0,016	0,136	0,892
	Ba_GMV	0,007	0,024	0,136	0,299	0,765
	Ce_GMV	0,037	0,037	0,105	1,006	0,316
	Co_GMV	0,075	0,097	0,122	0,777	0,438
	CAT_GMV	0,040	0,021	0,343	1,876	0,062
	La_GMV	0,006	0,006	0,151	0,957	0,340
	PL_GMV	-0,091	0,103	-0,208	-0,884	0,378
a. Dependent Variable: Angry hostility						
B = unstandardized beta; SE = standard error; β = standardized beta; t = t value; p = p value						

Model Summary					
**Model**	**R**	**R Square**	**Adjusted R Square**	**SE of the Estimate**	f^2
**1**	0,331[a]	0,110	0,065	4,749	0,124
a. Predictors: (Constant), Sex, Age, AAA_INVF, AB_INVF, Ba_INVF, Ce_INVF, Co_INVF, CAT_INVF, La_INVF, PL_INVF					
R = R value; SE = standard error; $f2$ = Cohen's $f2$					

ANOVA[a]						
**Model**		**Sum of Squares**	**df**	**Mean Square**	**F**	**p**
1	**Regression**	557,778	10,000	55,778	2,474	0,008[b]
	**Residual**	4532,226	201,000	22,548		
	**Total**	5090,004	211,000			
a. Dependent Variable: Angry hostility						
b. Predictors: (Constant), Sex, Age, AAA_INVF, AB_INVF, Ba_INVF, Ce_INVF, Co_INVF, CAT_INVF, La_INVF, PL_INVF						
df = degrees of freedom; F = F value; p = p value						

Coefficients[a]						
**Model**		**Unstandardized**		**Standardized Coefficients**	**t**	**p**
		**B**	**SE**	**β**		
1	**(Constant)**	31,900	8,262		3,861	0,000
	**Sex**	1,172	0,786	0,119	1,492	0,137
	**Age**	-0,074	0,103	-0,052	-0,717	0,474
	**AAA_INVF**	-17,554	25,723	-0,074	-0,682	0,496
	**AB_INVF**	-0,826	5,369	-0,011	-0,154	0,878
	**Ba_INVF**	7,223	33,116	0,024	0,218	0,828
	**Ce_INVF**	-0,159	6,687	-0,002	-0,024	0,981
	**Co_INVF**	-15,424	9,899	-0,118	-1,558	0,121
	**CAT_INVF**	2,238	3,855	0,040	0,581	0,562
	**La_INVF**	-36,217	24,174	-0,139	-1,498	0,136
	**PL_INVF**	6,014	5,225	0,079	1,151	0,251
a. Dependent Variable: Angry hostility						
B = unstandardized beta; SE = standard error; β = standardized beta; t = t value; p = p value						

Model Summary					
**Model**	**R**	**R Square**	**Adjusted R Square**	**SE of the Estimate**	f^2
**1**	0,379[a]	0,144	0,101	4,657	0,168
a. Predictors: (Constant), Sex, Age, AAA_ODI, AB_ODI, Ba_ODI, Ce_ODI, Co_ODI, CAT_ODI, La_ODI, PL_ODI					
R = R value; SE = standard error; $f2$ = Cohen's $f2$					

ANOVA[a]						
**Model**		**Sum of Squares**	**df**	**Mean Square**	**F**	**p**
1	**Regression**	730,735	10,000	73,073	3,369	0,000[b]
	**Residual**	4359,269	201,000	21,688		
	**Total**	5090,004	211,000			
a. Dependent Variable: Angry hostility						
b. Predictors: (Constant), Sex, Age, AAA_ODI, AB_ODI, Ba_ODI, Ce_ODI, Co_ODI, CAT_ODI, La_ODI, PL_ODI						
df = degrees of freedom; F = F value; p = p value						

Coefficients[a]						
**Model**		**Unstandardized Coefficients**		**Standardized Coefficients**	**t**	**p**
		**B**	**SE**	**β**		
1	**(Constant)**	18,469	6,818		2,709	0,007
	**Sex**	2,030	0,657	0,207	3,088	0,002
	**Age**	-0,172	0,096	-0,120	-1,780	0,077
	**AAA_ODI**	-5,649	13,731	-0,036	-0,411	0,681
	**AB_ODI**	3,871	3,093	0,090	1,252	0,212
	**Ba_ODI**	22,943	13,668	0,146	1,679	0,095
	**Ce_ODI**	1,315	5,173	0,018	0,254	0,800
	**Co_ODI**	-26,329	11,389	-0,186	-2,312	0,022
	**CAT_ODI**	15,787	5,666	0,187	2,786	0,006
	**La_ODI**	-18,020	9,423	-0,154	-1,912	0,057
	**PL_ODI**	4,987	2,771	0,121	1,800	0,073
a. Dependent Variable: Angry hostility						
B = unstandardized beta; SE = standard error; β = standardized beta; t = t value; p = p value						

Model Summary					
Model	R	R Square	Adjusted R Square	SE of the Estimate	f^2
1	0,319[a]	0,102	-1,181	7,254	0,114
a. Predictors: (Constant), Sex, Age, AAA_ISO, AB_ISO, Ba_ISO, Ce_ISO, Co_ISO, CAT_ISO, La_ISO, PL_ISO					
R = R value; SE = standard error; $f2$ = Cohen's $f2$					

ANOVA[a]						
Model		Sum of Squares	df	Mean Square	F	p
1	Regression	41,735	10,000	4,173	0,079	1,000[b]
	Residual	368,360	7,000	52,623		
	Total	410,095	17,000			
a. Dependent Variable: Angry hostility						
b. Predictors: (Constant), Sex, Age, AAA_ISO, AB_ISO, Ba_ISO, Ce_ISO, Co_ISO, CAT_ISO, La_ISO, PL_ISO						
df = degrees of freedom; F = F value; p = p value						

Coefficients[a]						
Model		Unstandardized Coefficients		Standardized Coefficients	t	p
		B	SE	β		
1	(Constant)	16,551	17,885		0,925	0,386
	Sex	2,148	3,659	0,219	0,587	0,576
	Age	-0,159	0,530	-0,111	-0,300	0,773
	AAA_ISO	-2,514	22,543	-0,048	-0,112	0,914
	AB_ISO	4,654	18,571	0,106	0,251	0,809
	Ba_ISO	1,164	22,397	0,022	0,052	0,960
	Ce_ISO	-1,481	13,244	-0,046	-0,112	0,914
	Co_ISO	-2,225	20,898	-0,042	-0,106	0,918
	CAT_ISO	4,047	13,649	0,110	0,296	0,775
	La_ISO	-4,154	20,844	-0,075	-0,199	0,848
	PL_ISO	6,548	26,772	0,103	0,245	0,814
a. Dependent Variable: Angry hostility						
B = unstandardized beta; SE = standard error; β = standardized beta; t = t value; p = p value						

Model Summary					
**Model**	**R**	**R Square**	**Adjusted R Square**	**SE of the Estimate**	f^2
**1**	0,200[a]	0,040	-0,006	5,888	0,042
a. Predictors: (Constant), Sex, Age, AAA_GMV, AB_GMV, Ba_GMV, Ce_GMV, Co_GMV, CAT_GMV, La_GMV, PL_GMV					
R = R value; SE = standard error; $f2$ = Cohen's $f2$					

ANOVA[a]						
**Model**		**Sum of Squares**	**df**	**Mean Square**	**F**	**p**
**1**	**Regression**	302,998	10,000	30,300	0,874	0,558[b]
	**Residual**	7280,984	210,000	34,671		
	**Total**	7583,982	220,000			
a. Dependent Variable: Depression						
b. Predictors: (Constant), Sex, Age, AAA_GMV, AB_GMV, Ba_GMV, Ce_GMV, Co_GMV, CAT_GMV, La_GMV, PL_GMV						
df = degrees of freedom; F = F value; p = p value						

Coefficients[a]						
**Model**		**Unstandardized Coefficients**		**Standardized Coefficients**	**t**	**p**
		**B**	**SE**	**β**		
**1**	**(Constant)**	5,925	6,081		0,974	0,331
	**Sex**	2,088	0,956	0,178	2,185	0,030
	**Age**	-0,076	0,119	-0,044	-0,638	0,524
	**AAA_GMV**	-0,020	0,038	-0,208	-0,523	0,601
	**AB_GMV**	0,029	0,049	0,072	0,591	0,555
	**Ba_GMV**	0,008	0,029	0,131	0,280	0,780
	**Ce_GMV**	0,017	0,046	0,040	0,372	0,711
	**Co_GMV**	0,038	0,119	0,052	0,324	0,747
	**CAT_GMV**	0,012	0,026	0,088	0,467	0,641
	**La_GMV**	-0,004	0,007	-0,083	-0,512	0,609
	**PL_GMV**	0,028	0,127	0,053	0,221	0,825
a. Dependent Variable: Depression						
B = unstandardized beta; SE = standard error; β = standardized beta; t = t value; p = p value						

Model Summary					
**Model**	**R**	**R Square**	**Adjusted R Square**	**SE of the Estimate**	f^2
**1**	0,318[a]	0,101	0,057	5,703	0,112
a. Predictors: (Constant), Sex, Age, AAA_INVF, AB_INVF, Ba_INVF, Ce_INVF, Co_INVF, CAT_INVF, La_INVF, PL_INVF					
R = R value; SE = standard error; $f2$ = Cohen's $f2$					

ANOVA[a]						
**Model**		**Sum of Squares**	**df**	**Mean Square**	**F**	**p**
**1**	**Regression**	736,381	10,000	73,638	2,264	0,016[b]
	**Residual**	6537,347	201,000	32,524		
	**Total**	7273,728	211,000			
a. Dependent Variable: Depression						
b. Predictors: (Constant), Sex, Age, AAA_INVF, AB_INVF, Ba_INVF, Ce_INVF, Co_INVF, CAT_INVF, La_INVF, PL_INVF						
df = degrees of freedom; F = F value; p = p value						

Coefficients[a]						
**Model**		**Unstandardized Coefficients**		**Standardized Coefficients**	**t**	**p**
		**B**	**SE**	**β**		
**1**	**(Constant)**	34,863	9,922		3,513	0,001
	**Sex**	0,283	0,944	0,024	0,300	0,764
	**Age**	-0,002	0,124	-0,001	-0,018	0,986
	**AAA_INVF**	4,445	30,894	0,016	0,144	0,886
	**AB_INVF**	0,450	6,448	0,005	0,070	0,944
	**Ba_INVF**	38,563	39,772	0,105	0,970	0,333
	**Ce_INVF**	3,547	8,031	0,033	0,442	0,659
	**Co_INVF**	-22,091	11,889	-0,141	-1,858	0,065
	**CAT_INVF**	0,109	4,629	0,002	0,023	0,981
	**La_INVF**	-98,108	29,033	-0,315	-3,379	0,001
	**PL_INVF**	7,162	6,275	0,079	1,141	0,255
a. Dependent Variable: Depression						
B = unstandardized beta; SE = standard error; β = standardized beta; t = t value; p = p value						

Model Summary					
**Model**	**R**	**R Square**	**Adjusted R Square**	**SE of the Estimate**	f^2
**1**	0,220[a]	0,048	0,001	5,869	0,050
a. Predictors: (Constant), Sex, Age, AAA_ODI, AB_ODI, Ba_ODI, Ce_ODI, Co_ODI, CAT_ODI, La_ODI, PL_ODI					
R = R value; SE = standard error; $f2$ = Cohen's $f2$					

ANOVA[a]						
**Model**		**Sum of Squares**	**df**	**Mean Square**	**F**	**p**
**1**	**Regression**	351,106	10,000	35,111	1,019	0,428[b]
	**Residual**	6922,623	201,000	34,441		
	**Total**	7273,728	211,000			
a. Dependent Variable: Depression						
b. Predictors: (Constant), Sex, Age, AAA_ODI, AB_ODI, Ba_ODI, Ce_ODI, Co_ODI, CAT_ODI, La_ODI, PL_ODI						
df = degrees of freedom; F = F value; p = p value						

Coefficients[a]						
**Model**		**Unstandardized Coefficients**		**Standardized Coefficients**	**t**	**p**
		**B**	**SE**	**β**		
**1**	**(Constant)**	13,727	8,592		1,598	0,112
	**Sex**	1,535	0,828	0,131	1,854	0,065
	**Age**	-0,096	0,122	-0,056	-0,791	0,430
	**AAA_ODI**	1,778	17,303	0,009	0,103	0,918
	**AB_ODI**	0,993	3,897	0,019	0,255	0,799
	**Ba_ODI**	6,459	17,224	0,034	0,375	0,708
	**Ce_ODI**	1,590	6,519	0,018	0,244	0,808
	**Co_ODI**	-5,893	14,353	-0,035	-0,411	0,682
	**CAT_ODI**	8,291	7,140	0,082	1,161	0,247
	**La_ODI**	-14,577	11,875	-0,104	-1,228	0,221
	**PL_ODI**	5,145	3,492	0,104	1,473	0,142
a. Dependent Variable: Depression						
B = unstandardized beta; SE = standard error; β = standardized beta; t = t value; p = p value						

Model Summary					
**Model**	**R**	**R Square**	**Adjusted R Square**	**SE of the Estimate**	f^2
**1**	0,292[a]	0,085	-1,221	8,751	0,093
a. Predictors: (Constant), Sex, Age, AAA_ISO, AB_ISO, Ba_ISO, Ce_ISO, Co_ISO, CAT_ISO, La_ISO, PL_ISO					
R = R value; SE = standard error; $f2$ = Cohen's $f2$					

ANOVA[a]						
**Model**		**Sum of Squares**	**df**	**Mean Square**	**F**	**p**
1	**Regression**	50,036	10,000	5,004	0,065	1,000[b]
	**Residual**	535,999	7,000	76,571		
	**Total**	586,035	17,000			
a. Dependent Variable: Depression						
b. Predictors: (Constant), Sex, Age, AAA_ISO, AB_ISO, Ba_ISO, Ce_ISO, Co_ISO, CAT_ISO, La_ISO, PL_ISO						
df = degrees of freedom; F = F value; p = p value						

Coefficients[a]						
**Model**		**Unstandardized Coefficients**		**Standardized Coefficients**	**t**	**p**
		**B**	**SE**	**β**		
1	**(Constant)**	20,038	21,575		0,929	0,384
	**Sex**	1,827	4,414	0,156	0,414	0,691
	**Age**	-0,047	0,639	-0,027	-0,073	0,944
	**AAA_ISO**	1,224	27,193	0,019	0,045	0,965
	**AB_ISO**	0,165	22,402	0,003	0,007	0,994
	**Ba_ISO**	-5,231	27,016	-0,083	-0,194	0,852
	**Ce_ISO**	0,440	15,976	0,011	0,028	0,979
	**Co_ISO**	-4,226	25,208	-0,067	-0,168	0,872
	**CAT_ISO**	2,573	16,464	0,059	0,156	0,880
	**La_ISO**	-11,439	25,144	-0,172	-0,455	0,663
	**PL_ISO**	-12,802	32,294	-0,168	-0,396	0,704
a. Dependent Variable: Depression						
B = unstandardized beta; SE = standard error; β = standardized beta; t = t value; p = p value						

Model Summary					
**Model**	**R**	**R Square**	**Adjusted R Square**	**SE of the Estimate**	f^2
**1**	0,249[a]	0,062	0,017	4,786	0,066
a. Predictors: (Constant), Sex, Age, AAA_GMV, AB_GMV, Ba_GMV, Ce_GMV, Co_GMV, CAT_GMV, La_GMV, PL_GMV					
R = R value; SE = standard error; $f2$ = Cohen's $f2$					

ANOVA[a]						
**Model**		**Sum of Squares**	**df**	**Mean Square**	**F**	**p**
**1**	**Regression**	318,156	10,000	31,816	1,389	0,187[b]
	**Residual**	4810,604	210,000	22,908		
	**Total**	5128,760	220,000			
a. Dependent Variable: Self-Consciousness						
b. Predictors: (Constant), Sex, Age, AAA_GMV, AB_GMV, Ba_GMV, Ce_GMV, Co_GMV, CAT_GMV, La_GMV, PL_GMV						
df = degrees of freedom; F = F value; p = p value						

Coefficients[a]						
**Model**		**Unstandardized Coefficients**		**Standardized Coefficients**	**t**	**p**
		**B**	**SE**	**β**		
**1**	**(Constant)**	11,814	4,943		2,390	0,018
	**Sex**	2,455	0,777	0,255	3,160	0,002
	**Age**	0,034	0,097	0,024	0,350	0,727
	**AAA_GMV**	0,003	0,031	0,032	0,081	0,936
	**AB_GMV**	0,022	0,040	0,064	0,535	0,593
	**Ba_GMV**	-0,021	0,024	-0,404	-0,875	0,383
	**Ce_GMV**	0,020	0,037	0,057	0,535	0,594
	**Co_GMV**	0,052	0,097	0,086	0,542	0,588
	**CAT_GMV**	0,002	0,021	0,020	0,107	0,915
	**La_GMV**	-0,001	0,006	-0,027	-0,169	0,866
	**PL_GMV**	0,127	0,103	0,294	1,232	0,219
a. Dependent Variable: Self-Consciousness						
B = unstandardized beta; SE = standard error; β = standardized beta; t = t value; p = p value						

Model Summary					
Model	R	R Square	Adjusted R Square	SE of the Estimate	f^2
1	0,257[a]	0,066	0,019	4,781	0,071

a. Predictors: (Constant), Sex, Age, AAA_INVF, AB_INVF, Ba_INVF, Ce_INVF, Co_INVF, CAT_INVF, La_INVF, PL_INVF

R = R value; SE = standard error; $f2$ = Cohen's $f2$

ANOVA[a]						
Model		Sum of Squares	df	Mean Square	F	p
1	Regression	324,399	10,000	32,440	1,419	0,174[b]
	Residual	4594,548	201,000	22,858		
	Total	4918,947	211,000			

a. Dependent Variable: Self-consciousness

b. Predictors: (Constant), Sex, Age, AAA_INVF, AB_INVF, Ba_INVF, Ce_INVF, Co_INVF, CAT_INVF, La_INVF, PL_INVF

df = degrees of freedom; F = F value; p = p value

Coefficients[a]						
Model		Unstandardized Coefficients		Standardized Coefficients	t	p
		B	SE	β		
1	(Constant)	19,921	8,318		2,395	0,018
	Sex	1,924	0,791	0,199	2,432	0,016
	Age	0,071	0,104	0,050	0,682	0,496
	AAA_INVF	-6,381	25,899	-0,027	-0,246	0,806
	AB_INVF	5,390	5,406	0,071	0,997	0,320
	Ba_INVF	13,692	33,343	0,045	0,411	0,682
	Ce_INVF	0,250	6,733	0,003	0,037	0,970
	Co_INVF	-3,813	9,967	-0,030	-0,383	0,702
	CAT_INVF	2,461	3,881	0,045	0,634	0,527
	La_INVF	-26,171	24,340	-0,102	-1,075	0,284
	PL_INVF	-2,614	5,260	-0,035	-0,497	0,620

a. Dependent Variable: Self-consiousness

B = unstandardized beta; SE = standard error; β = standardized beta; t = t value; p = p value

Model Summary					
**Model**	**R**	**R Square**	**Adjusted R Square**	**SE of the Estimate**	f^2
**1**	0,299[a]	0,090	0,044	4,720	0,099
a. Predictors: (Constant), Sex, Age, AAA_ODI, AB_ODI, Ba_ODI, Ce_ODI, Co_ODI, CAT_ODI, La_ODI, PL_ODI					
R = R value; SE = standard error; $f2$ = Cohen's $f2$					

ANOVA[a]						
**Model**		**Sum of Squares**	**df**	**Mean Square**	**F**	**p**
**1**	**Regression**	440,549	10,000	44,055	1,977	0,037[b]
	**Residual**	4478,398	201,000	22,281		
	**Total**	4918,947	211,000			
a. Dependent Variable: Self-consiousness						
b. Predictors: (Constant), Sex, Age, AAA_ODI, AB_ODI, Ba_ODI, Ce_ODI, Co_ODI, CAT_ODI, La_ODI, PL_ODI						
df = degrees of freedom; F = F value; p = p value						

Coefficients[a]						
**Model**		**Unstandardized Coefficients**		**Standardized Coefficients**	**t**	**p**
		**B**	**SE**	**β**		
**1**	**(Constant)**	21,055	6,910		3,047	0,003
	**Sex**	2,058	0,666	0,213	3,089	0,002
	**Age**	0,039	0,098	0,027	0,396	0,693
	**AAA_ODI**	-6,021	13,917	-0,039	-0,433	0,666
	**AB_ODI**	2,198	3,135	0,052	0,701	0,484
	**Ba_ODI**	20,645	13,854	0,134	1,490	0,138
	**Ce_ODI**	0,446	5,244	0,006	0,085	0,932
	**Co_ODI**	-15,626	11,544	-0,112	-1,354	0,177
	**CAT_ODI**	4,081	5,743	0,049	0,711	0,478
	**La_ODI**	-24,575	9,551	-0,214	-2,573	0,011
	**PL_ODI**	2,860	2,808	0,071	1,018	0,310
a. Dependent Variable: Self-consciousness						
B = unstandardized beta; SE = standard error; β = standardized beta; t = t value; p = p value						

Model Summary					
Model	R	R Square	Adjusted R Square	SE of the Estimate	f^2
1	0,296[a]	0,088	-1,216	7,187	0,096
a. Predictors: (Constant), Sex, Age, AAA_ISO, AB_ISO, Ba_ISO, Ce_ISO, Co_ISO, CAT_ISO, La_ISO, PL_ISO					
R = R value; SE = standard error; $f2$ = Cohen's $f2$					

ANOVA[a]						
Model		Sum of Squares	df	Mean Square	F	p
1	Regression	34,716	10,000	3,472	0,067	1,000[b]
	Residual	361,597	7,000	51,657		
	Total	396,313	17,000			
a. Dependent Variable: Self-consciousness						
b. Predictors: (Constant), Sex, Age, AAA_ISO, AB_ISO, Ba_ISO, Ce_ISO, Co_ISO, CAT_ISO, La_ISO, PL_ISO						
df = degrees of freedom; F = F value; p = p value						

Coefficients[a]						
Model		Unstandardized Coefficients		Standardized Coefficients	t	p
		B	SE	β		
1	(Constant)	18,431	17,720		1,040	0,333
	Sex	2,264	3,626	0,235	0,624	0,552
	Age	0,076	0,525	0,054	0,144	0,890
	AAA_ISO	2,020	22,335	0,039	0,090	0,930
	AB_ISO	1,009	18,400	0,023	0,055	0,958
	Ba_ISO	-3,681	22,190	-0,071	-0,166	0,873
	Ce_ISO	-0,508	13,122	-0,016	-0,039	0,970
	Co_ISO	-2,266	20,705	-0,044	-0,109	0,916
	CAT_ISO	0,829	13,523	0,023	0,061	0,953
	La_ISO	-8,289	20,652	-0,152	-0,401	0,700
	PL_ISO	-6,227	26,525	-0,099	-0,235	0,821
a. Dependent Variable: Self-consciousness						
B = unstandardized beta; SE = standard error; β = standardized beta; t = t value; p = p value						

Model Summary					
**Model**	**R**	**R Square**	**Adjusted R Square**	**SE of the Estimate**	f^2
**1**	0,224[a]	0,050	0,005	4,443	0,053
a. Predictors: (Constant), Sex, Age, AAA_GMV, AB_GMV, Ba_GMV, Ce_GMV, Co_GMV, CAT_GMV, La_GMV, PL_GMV					
R = R value; SE = standard error; $f2$ = Cohen's $f2$					

ANOVA[a]						
**Model**		**Sum of Squares**	**df**	**Mean Square**	**F**	**p**
**1**	**Regression**	219,951	10,000	21,995	1,114	0,353[b]
	**Residual**	4145,053	210,000	19,738		
	**Total**	4365,005	220,000			
a. Dependent Variable: Impulsivity						
b. Predictors: (Constant), Sex, Age, AAA_GMV, AB_GMV, Ba_GMV, Ce_GMV, Co_GMV, CAT_GMV, La_GMV, PL_GMV						
df = degrees of freedom; F = F value; p = p value						

Coefficients[a]						
**Model**		**Unstandardized Coefficients**		**Standardized Coefficients**	**t**	**p**
		**B**	**SE**	**β**		
**1**	**(Constant)**	19,354	4,589		4,218	0,000
	**Sex**	1,232	0,721	0,139	1,708	0,089
	**Age**	-0,178	0,090	-0,137	-1,984	0,049
	**AAA_GMV**	-0,025	0,029	-0,338	-0,857	0,393
	**AB_GMV**	0,019	0,037	0,061	0,506	0,613
	**Ba_GMV**	0,008	0,022	0,178	0,383	0,702
	**Ce_GMV**	-0,002	0,034	-0,008	-0,071	0,944
	**Co_GMV**	0,068	0,090	0,121	0,756	0,450
	**CAT_GMV**	0,003	0,020	0,025	0,136	0,892
	**La_GMV**	0,001	0,006	0,041	0,255	0,799
	**PL_GMV**	-0,014	0,096	-0,035	-0,144	0,886
a. Dependent Variable: Impulsivity						
B = unstandardized beta; SE = standard error; β = standardized beta; t = t value; p = p value						

Model Summary					
**Model**	**R**	**R Square**	**Adjusted R Square**	**SE of the Estimate**	f^2
**1**	0,344[a]	0,119	0,075	4,285	0,135
a. Predictors: (Constant), Sex, Age, AAA_INVF, AB_INVF, Ba_INVF, Ce_INVF, Co_INVF, CAT_INVF, La_INVF, PL_INVF					
R = R value; SE = standard error; $f2$ = Cohen's $f2$					

ANOVA[a]						
**Model**		**Sum of Squares**	**df**	**Mean Square**	**F**	**p**
**1**	**Regression**	496,397	10,000	49,640	2,704	0,004[b]
	**Residual**	3690,039	201,000	18,358		
	**Total**	4186,436	211,000			
a. Dependent Variable: Impulsivity						
b. Predictors: (Constant), Sex, Age, AAA_INVF, AB_INVF, Ba_INVF, Ce_INVF, Co_INVF, CAT_INVF, La_INVF, PL_INVF						
df = degrees of freedom; F = F value; p = p value						

Coefficients[a]						
**Model**		**Unstandardized Coefficients**		**Standardized Coefficients**	**t**	**p**
		**B**	**SE**	**β**		
**1**	**(Constant)**	32,968	7,455		4,422	0,000
	**Sex**	0,545	0,709	0,061	0,768	0,443
	**Age**	-0,101	0,093	-0,078	-1,088	0,278
	**AAA_INVF**	-54,676	23,210	-0,255	-2,356	0,019
	**AB_INVF**	5,277	4,845	0,076	1,089	0,277
	**Ba_INVF**	15,436	29,881	0,055	0,517	0,606
	**Ce_INVF**	8,799	6,034	0,109	1,458	0,146
	**Co_INVF**	-5,907	8,932	-0,050	-0,661	0,509
	**CAT_INVF**	0,365	3,478	0,007	0,105	0,916
	**La_INVF**	-20,628	21,813	-0,087	-0,946	0,345
	**PL_INVF**	8,833	4,714	0,128	1,874	0,062
a. Dependent Variable: Impulsivity						
B = unstandardized beta; SE = standard error; β = standardized beta; t = t value; p = p value						

Model Summary					
**Model**	**R**	**R Square**	**Adjusted R Square**	**SE of the Estimate**	f^2
**1**	0,255[a]	0,065	0,019	4,413	0,070
a. Predictors: (Constant), Sex, Age, AAA_ODI, AB_ODI, Ba_ODI, Ce_ODI, Co_ODI, CAT_ODI, La_ODI, PL_ODI					
R = R value; SE = standard error; $f2$ = Cohen's $f2$					

ANOVA[a]						
**Model**		**Sum of Squares**	**df**	**Mean Square**	**F**	**p**
**1**	**Regression**	272,603	10,000	27,260	1,400	0,182[b]
	**Residual**	3913,833	201,000	19,472		
	**Total**	4186,436	211,000			
a. Dependent Variable: Impulsivity						
a. Predictors: (Constant), Sex, Age, AAA_ODI, AB_ODI, Ba_ODI, Ce_ODI, Co_ODI, CAT_ODI, La_ODI, PL_ODI						
df = degrees of freedom; F = F value; p = p value						

Coefficients[a]						
**Model**		**Unstandardized Coefficients**		**Standardized Coefficients**	**t**	**p**
		**B**	**SE**	**β**		
**1**	**(Constant)**	19,554	6,460		3,027	0,003
	**Sex**	1,218	0,623	0,137	1,956	0,052
	**Age**	-0,175	0,091	-0,135	-1,920	0,056
	**AAA_ODI**	-6,128	13,011	-0,043	-0,471	0,638
	**AB_ODI**	1,026	2,930	0,026	0,350	0,727
	**Ba_ODI**	4,003	12,951	0,028	0,309	0,758
	**Ce_ODI**	-0,463	4,902	-0,007	-0,094	0,925
	**Co_ODI**	3,668	10,792	0,029	0,340	0,734
	**CAT_ODI**	4,406	5,369	0,058	0,821	0,413
	**La_ODI**	-6,436	8,929	-0,061	-0,721	0,472
	**PL_ODI**	4,657	2,625	0,124	1,774	0,078
a. Dependent Variable: Impulsivity						
B = unstandardized beta; SE = standard error; β = standardized beta; t = t value; p = p value						

Model Summary					
**Model**	**R**	**R Square**	**Adjusted R Square**	**SE of the Estimate**	f^2
**1**	0,240[a]	0,058	-1,288	6,738	0,062
a. Predictors: (Constant), Sex, Age, AAA_ISO, AB_ISO, Ba_ISO, Ce_ISO, Co_ISO, CAT_ISO, La_ISO, PL_ISO					
R = R value; SE = standard error; $f2$ = Cohen's $f2$					

ANOVA[a]						
**Model**		**Sum of Squares**	**df**	**Mean Square**	**F**	**p**
**1**	**Regression**	19,479	10,000	1,948	0,043	1,000[b]
	**Residual**	317,817	7,000	45,402		
	**Total**	337,296	17,000			
a. Dependent Variable: Impulsivity						
b. Predictors: (Constant), Sex, Age, AAA_ISO, AB_ISO, Ba_ISO, Ce_ISO, Co_ISO, CAT_ISO, La_ISO, PL_ISO						
df = degrees of freedom; F = F value; p = p value						

Coefficients[a]						
**Model**		**Unstandardized Coefficients**		**Standardized Coefficients**	**t**	**p**
		**B**	**SE**	**β**		
**1**	**(Constant)**	24,129	16,613		1,452	0,190
	**Sex**	1,276	3,399	0,143	0,376	0,718
	**Age**	-0,172	0,492	-0,132	-0,348	0,738
	**AAA_ISO**	-3,321	20,939	-0,069	-0,159	0,878
	**AB_ISO**	-0,292	17,250	-0,007	-0,017	0,987
	**Ba_ISO**	-1,186	20,803	-0,025	-0,057	0,956
	**Ce_ISO**	1,722	12,302	0,059	0,140	0,893
	**Co_ISO**	-1,191	19,411	-0,025	-0,061	0,953
	**CAT_ISO**	-0,313	12,678	-0,009	-0,025	0,981
	**La_ISO**	-4,078	19,362	-0,081	-0,211	0,839
	**PL_ISO**	-5,306	24,868	-0,092	-0,213	0,837
a. Dependent Variable: Impulsivity						
B = unstandardized beta; SE = standard error; β = standardized beta; t = t value; p = p value						

Model Summary					
Model	R	R Square	Adjusted R Square	SE of the Estimate	f^2
1	0,279[a]	0,078	0,034	4,631	0,085
a. Predictors: (Constant), Sex, Age, AAA_GMV, AB_GMV, Ba_GMV, Ce_GMV, Co_GMV, CAT_GMV, La_GMV, PL_GMV					
R = R value; SE = standard error; $f2$ = Cohen's $f2$					

ANOVA[a]						
Model		Sum of Squares	df	Mean Square	F	p
1	Regression	380,930	10,000	38,093	1,776	0,067[b]
	Residual	4504,617	210,000	21,451		
	Total	4885,548	220,000			
a. Dependent Variable: Vulnerability						
b. Predictors: (Constant), Sex, Age, AAA_GMV, AB_GMV, Ba_GMV, Ce_GMV, Co_GMV, CAT_GMV, La_GMV, PL_GMV						
df = degrees of freedom; F = F value; p = p value						

Coefficients[a]						
Model		Unstandardized Coefficients		Standardized Coefficients	t	p
		B	SE	β		
1	(Constant)	7,243	4,783		1,514	0,132
	Sex	2,608	0,752	0,277	3,468	0,001
	Age	-0,123	0,094	-0,089	-1,311	0,191
	AAA_GMV	0,004	0,030	0,047	0,120	0,905
	AB_GMV	0,017	0,039	0,053	0,448	0,655
	Ba_GMV	-0,028	0,023	-0,560	-1,220	0,224
	Ce_GMV	0,009	0,036	0,027	0,255	0,799
	Co_GMV	-0,012	0,093	-0,020	-0,128	0,898
	CAT_GMV	0,013	0,020	0,116	0,628	0,531
	La_GMV	0,006	0,006	0,169	1,064	0,288
	PL_GMV	0,133	0,100	0,314	1,327	0,186
a. Dependent Variable: Vulnerability						
B = unstandardized beta; SE = standard error; β = standardized beta; t = t value; p = p value						

Model Summary					
**Model**	**R**	**R Square**	**Adjusted R Square**	**SE of the Estimate**	f^2
**1**	0,325[a]	0,105	0,061	4,567	0,117
a. Predictors: (Constant), Sex, Age, AAA_INVF, AB_INVF, Ba_INVF, Ce_INVF, Co_INVF, CAT_INVF, La_INVF, PL_INVF					
R = R value; SE = standard error; $f2$ = Cohen's $f2$					

ANOVA[a]						
**Model**		**Sum of Squares**	**df**	**Mean Square**	**F**	**p**
**1**	**Regression**	494,062	10,000	49,406	2,369	0,011[b]
	**Residual**	4191,622	201,000	20,854		
	**Total**	4685,684	211,000			
a. Dependent Variable: Vulnerability						
b. Predictors: (Constant), Sex, Age, AAA_INVF, AB_INVF, Ba_INVF, Ce_INVF, Co_INVF, CAT_INVF, La_INVF, PL_INVF						
df = degrees of freedom; F = F value; p = p value						

Coefficients[a]						
**Model**		**Unstandardized Coefficients**		**Standardized Coefficients**	**t**	**p**
		**B**	**SE**	**β**		
**1**	**(Constant)**	28,281	7,945		3,559	0,000
	**Sex**	1,036	0,756	0,110	1,371	0,172
	**Age**	-0,054	0,099	-0,039	-0,540	0,590
	**AAA_INVF**	20,051	24,738	0,088	0,811	0,419
	**AB_INVF**	0,814	5,163	0,011	0,158	0,875
	**Ba_INVF**	8,950	31,847	0,030	0,281	0,779
	**Ce_INVF**	-5,542	6,431	-0,065	-0,862	0,390
	**Co_INVF**	-13,387	9,520	-0,107	-1,406	0,161
	**CAT_INVF**	3,541	3,707	0,066	0,955	0,341
	**La_INVF**	-64,634	23,248	-0,259	-2,780	0,006
	**PL_INVF**	3,696	5,025	0,051	0,736	0,463
a. Dependent Variable: Vulnerability						
B = unstandardized beta; SE = standard error; β = standardized beta; t = t value; p = p value						

Model Summary					
**Model**	**R**	**R Square**	**Adjusted R Square**	**SE of the Estimate**	f^2
**1**	0,314[a]	0,099	0,054	4,584	0,110
a. Predictors: (Constant), Sex, Age, AAA_ODI, AB_ODI, Ba_ODI, Ce_ODI, Co_ODI, CAT_ODI, La_ODI, PL_ODI					
R = R value; SE = standard error; $f2$ = Cohen's $f2$					

ANOVA[a]						
**Model**		**Sum of Squares**	**df**	**Mean Square**	**F**	**p**
**1**	**Regression**	462,635	10,000	46,263	2,202	0,019[b]
	**Residual**	4223,049	201,000	21,010		
	**Total**	4685,684	211,000			
a. Dependent Variable: Vulnerability						
b. Predictors: (Constant), Sex, Age, AAA_ODI, AB_ODI, Ba_ODI, Ce_ODI, Co_ODI, CAT_ODI, La_ODI, PL_ODI						
df = degrees of freedom; F = F value; p = p value						

Coefficients[a]						
**Model**		**Unstandardized Coefficients**		**Standardized Coefficients**	**t**	**p**
		**B**	**SE**	**β**		
**1**	**(Constant)**	19,163	6,711		2,856	0,005
	**Sex**	1,837	0,647	0,195	2,839	0,005
	**Age**	-0,117	0,095	-0,085	-1,231	0,220
	**AAA_ODI**	-9,997	13,515	-0,066	-0,740	0,460
	**AB_ODI**	-0,258	3,044	-0,006	-0,085	0,933
	**Ba_ODI**	16,704	13,453	0,111	1,242	0,216
	**Ce_ODI**	-3,943	5,092	-0,056	-0,774	0,440
	**Co_ODI**	-10,260	11,210	-0,076	-0,915	0,361
	**CAT_ODI**	8,317	5,577	0,103	1,491	0,137
	**La_ODI**	-17,034	9,275	-0,152	-1,837	0,068
	**PL_ODI**	3,528	2,727	0,089	1,293	0,197
a. Dependent Variable: Vulnerability						
B = unstandardized beta; SE = standard error; β = standardized beta; t = t value; p = p value						

Model Summary					
**Model**	**R**	**R Square**	**Adjusted R Square**	**SE of the Estimate**	f^2
**1**	0,359[a]	0,129	-1,116	6,855	0,148
a. Predictors: (Constant), Sex, Age, AAA_ISO, AB_ISO, Ba_ISO, Ce_ISO, Co_ISO, CAT_ISO, La_ISO, PL_ISO					
R = R value; SE = standard error; $f2$ = Cohen's $f2$					

ANOVA[a]						
**Model**		**Sum of Squares**	**df**	**Mean Square**	**F**	**p**
**1**	**Regression**	48,564	10,000	4,856	0,103	0,999[b]
	**Residual**	328,956	7,000	46,994		
	**Total**	377,520	17,000			
a. Dependent Variable: Vulnerability						
b. Predictors: (Constant), Sex, Age, AAA_ISO, AB_ISO, Ba_ISO, Ce_ISO, Co_ISO, CAT_ISO, La_ISO, PL_ISO						
df = degrees of freedom; F = F value; p = p value						

Coefficients[a]						
**Model**		**Unstandardized Coefficients**		**Standardized Coefficients**	**t**	**p**
		**B**	**SE**	**β**		
**1**	**(Constant)**	15,968	16,902		0,945	0,376
	**Sex**	1,960	3,458	0,208	0,567	0,589
	**Age**	-0,049	0,501	-0,035	-0,097	0,925
	**AAA_ISO**	5,833	21,303	0,115	0,274	0,792
	**AB_ISO**	-4,297	17,550	-0,102	-0,245	0,814
	**Ba_ISO**	-3,991	21,165	-0,079	-0,189	0,856
	**Ce_ISO**	-4,563	12,516	-0,149	-0,365	0,726
	**Co_ISO**	0,728	19,748	0,014	0,037	0,972
	**CAT_ISO**	0,108	12,898	0,003	0,008	0,994
	**La_ISO**	-8,893	19,698	-0,167	-0,451	0,665
	**PL_ISO**	-8,100	25,300	-0,132	-0,320	0,758
a. Dependent Variable: Vulnerability						
B = unstandardized beta; SE = standard error; β = standardized beta; t = t value; p = p value						

Korrelationen										
**Controlvariables**			**AAA_INFV**	**AB_INVF**	**Ba_INVF**	**Ce_INVF**	**Co_INVF**	**CAT_INVF**	**La_INVF**	**Pl_INFV**
**Sex, Age**	**AAA_GVM**	**r**	-0,022	-0,060	0,081	0,086	0,102	0,155	-0,128	0,172
		**p**	0,743	0,376	0,232	0,204	0,133	0,024	0,058	0,011
		**df**	217	216	217	217	217	209	217	216
	**AB_GMV**	**r**	0,049	0,040	0,073	0,120	0,190	0,047	-0,012	0,059
		**p**	0,467	0,557	0,283	0,077	0,005	0,498	0,863	0,387
		**df**	217	216	217	217	217	209	217	216
	**Ba_GMV**	**r**	0,038	0,024	0,050	0,047	0,191	0,098	-0,124	0,131
		**p**	0,573	0,730	0,459	0,485	0,004	0,155	0,067	0,053
		**df**	217	216	217	217	217	209	217	216
	**Ce_GMV**	**r**	-0,021	-0,056	0,034	0,248	0,113	0,169	-0,024	0,058
		**p**	0,763	0,410	0,621	0,000	0,096	0,014	0,726	0,396
		**df**	217	216	217	217	217	209	217	216
	**Co_GMV**	**r**	-0,084	-0,107	0,078	0,036	-0,015	0,211	-0,133	0,173
		**p**	0,218	0,116	0,249	0,596	0,825	0,129	0,050	0,010
		**df**	217	216	217	217	217	209	217	216
	**CAT_GMV**	**r**	0,045	-0,008	0,089	0,032	0,099	0,084	-0,054	0,166
		**p**	0,511	0,906	0,190	0,641	0,143	0,223	0,428	0,014
		**df**	217	216	217	217	217	209	217	216
	**La_GMV**	**r**	0,092	-0,002	0,045	0,043	0,264	0,057	0,004	0,123
		**p**	0,174	0,980	0,506	0,524	0,000	0,410	0,953	0,069
		**df**	217	216	217	217	217	209	217	216
	**Pl_GMV**	**r**	0,052	0,054	-0,017	-0,034	0,194	0,076	-0,100	0,112
		**p**	0,444	0,425	0,797	0,614	0,004	0,271	0,139	0,100
		**df**	217	216	217	217	217	209	217	216
r= correlation coefficient; p = p value; df = degree of freedom										

Korrelationen										
Controlvariables			AAA_ODI	AB_ODI	Ba_ODI	Ce_ODI	Co_ODI	CAT_ODI	La_ODI	Pl_ODI
Sex, Age	AAA_GVM	r	0,178	-0,039	-0,087	0,194	0,140	0,017	-0,139	0,073
		p	0,008	0,562	0,198	0,004	0,038	0,804	0,039	0,281
		df	217	216	217	217	217	209	217	216
	AB_GMV	r	0,079	0,078	-0,050	0,137	0,105	-0,013	-0,093	0,023
		p	0,241	0,250	0,459	0,043	0,122	0,850	0,172	0,739
		df	217	216	217	217	217	209	217	216
	Ba_GMV	r	0,140	0,021	-0,078	0,130	0,209	-0,055	-0,212	0,038
		p	0,038	0,753	0,252	0,055	0,002	0,425	0,002	0,575
		df	217	216	217	217	217	209	217	216
	Ce_GMV	r	-0,031	-0,009	-0,069	0,136	0,083	-0,053	-0,139	0,013
		p	0,646	0,894	0,307	0,044	0,222	0,443	0,040	0,852
		df	217	216	217	217	217	209	217	216
	Co_GMV	r	0,173	-0,082	-0,087	0,192	-0,030	0,056	-0,037	0,078
		p	0,010	0,231	0,199	0,004	0,659	0,416	0,589	0,252
		df	217	216	217	217	217	209	217	216
	CAT_GMV	r	0,046	-0,032	-0,128	0,080	0,103	0,003	-0,151	0,096
		p	0,503	0,638	0,059	0,241	0,130	0,963	0,026	0,160
		df	217	216	217	217	217	209	217	216
	La_GMV	r	0,047	-0,020	-0,145	0,080	0,119	-0,065	-0,242	0,028
		p	0,491	0,766	0,032	0,238	0,079	0,348	0,000	0,677
		df	217	216	217	217	217	209	217	216
	Pl_GMV	r	0,063	0,000	-0,116	0,024	0,208	-0,103	-0,267	0,017
		p	0,351	1,000	0,087	0,728	0,002	0,136	0,000	0,800
		df	217	216	217	217	217	209	217	216
r= correlation coefficient; p = p value; df = degree of freedom										
